# Poly(ADP-ribose) Polymerase 1 (PARP1) restrains MyoD-dependent gene expression during muscle differentiation

**DOI:** 10.1038/s41598-020-72155-8

**Published:** 2020-09-15

**Authors:** Francesca Matteini, Oriella Andresini, Stefano Petrai, Cecilia Battistelli, Marianna Nicoletta Rossi, Rossella Maione

**Affiliations:** 1 Institut D’Investigació Biomèdica de Bellvitge (IDIBELL), P-CMR[C], Hospital Duran I Reynals Gran Via de L’Hospitalet, 199-203, L’Hospitalet de Llobregat, 08908 Barcelona, Spain; 2 Department of Molecular Medicine, Sapienza University of Rome, Viale Regina Elena 324, 00161 Rome, Italy; 3 Rheumatology Unit, Bambino Gesu Children’s Hospital (IRCCS), Viale di S. Paolo 15, 00146 Rome, Italy

**Keywords:** PolyADP-ribosylation, Differentiation, Epigenetics, Transcriptional regulatory elements

## Abstract

The myogenic factor MyoD regulates skeletal muscle differentiation by interacting with a variety of chromatin-modifying complexes. Although MyoD can induce and maintain chromatin accessibility at its target genes, its binding and trans-activation ability can be limited by some types of not fully characterized epigenetic constraints. In this work we analysed the role of PARP1 in regulating MyoD-dependent gene expression. PARP1 is a chromatin-associated enzyme, playing a well recognized role in DNA repair and that is implicated in transcriptional regulation. PARP1 affects gene expression through multiple mechanisms, often involving the Poly(ADP-ribosyl)ation of chromatin proteins. In line with PARP1 down-regulation during differentiation, we observed that PARP1 depletion boosts the up-regulation of MyoD targets, such as *p57*, *myogenin*, *Mef2C* and *p21*, while its re-expression reverts this effect. We also found that PARP1 interacts with some MyoD-binding regions and that its presence, independently of the enzymatic activity, interferes with MyoD recruitment and gene induction. We finally suggest a relationship between the binding of PARP1 and the loss of the activating histone modification H3K4me3 at MyoD-binding regions. This work highlights not only a novel player in the epigenetic control of myogenesis, but also a repressive and catalytic-independent mechanisms by which PARP1 regulates transcription.

## Introduction

Skeletal muscle differentiation is determined by the activation of a complex program of gene expression, which involves the temporally regulated induction of muscle-specific genes as well as of cell cycle inhibitors that ensure cell cycle exit of differentiating cells^1^. The entire process is promoted by MyoD, a muscle-specific transcription factor recognized as the master regulator of myogenesis, due to its capability of converting several non-muscle cell types to skeletal muscle cells^2^. MyoD regulates gene expression through a number of different and interdependent mechanisms, which are being increasingly discovered in the last years^3,4^. MyoD is a bHLH protein and interacts with most of its transcriptional targets by recognizing E-box sequences^5^. The induction of transcription by MyoD relies on its cooperation with other transcription factors, both ubiquitous and muscle-specific, and on its ability to interact with chromatin modifying enzymes^1^. The best established mechanisms by which the myogenic factor creates a chromatin environment permissive for transcription involves the recruitment of the histone acetylases p300/CBP and pCAF^6–8^ and the chromatin remodelling complex SWI/SNF^9,10^ on the targeted regulatory-regions. Moreover, MyoD functionally interacts with the arginine methyltransferase Prmt5^11^, with the lysine methyltransferase Set7/9^12^, with the chromatin remodelling enzyme Chd2^13^ and, as more recently reported, with the regulatory Long noncoding RNAs Linc-RAM^14^ and Kcnq1ot1^15^. MyoD has been shown to modify the chromatin status not only at promoters and enhancers, but also at intergenic regions^6^. In this regard, using the *p57*/*KvDMR1* locus ^16^ as a model, we provided evidence that MyoD can regulate gene expression from a distance by releasing repressive chromatin loops^17–19^. More recently, a direct role of MyoD in reorganizing the three-dimensional chromatin interactome during the myogenic conversion, has been reported^20^.

Although MyoD can initiate chromatin remodeling and histone modifications at target sites in heterochromatin, nevertheless its binding and transactivation capability are limited by some types of epigenetic constraints. In this regard, the poor ability of MyoD to convert some cell types to the muscle lineage has been ascribed, at least in part, to pre-existing chromatin features that preclude MyoD access to its targets^5,21^. For example, it has been reported that trimethylation of lysine 27 on histone H3 (H3K27me3) at the regulatory regions of certain muscle-specific genes prevents MyoD binding and gene activation in undifferentiated myoblasts. Upon differentiation stimuli, the recruitment of MyoD is enabled by the reduction of H3K27me3 levels, due to the down-regulation of EZH2, the histone methyltransferase that catalyzes this modification^22^. Similarly, the access of MyoD to a number of differentiation genes, before the onset of differentiation, is blocked by a repressor complex containing Snail and the histone deacetylases I and II, which is removed only upon differentiation stimuli^23^. The existence of epigenetic barriers for MyoD binding, involving EZH2 and H3K27me3, has also been suggested to contribute to the defective function of the myogenic factor in rhabdomyosarcoma cells^24^. Moreover, we have recently shown that accumulation of H3 lysine 9 dimethylation (H3K9me2) at a critical regulatory region of the MyoD target *p57kip2*, hinders the binding of MyoD and the consequent reorganization of the chromatin architecture, thus preventing the up-regulation of the gene in muscle cell types un-responsive for *p57* induction^17^. In light of the complexity of epigenetic regulation of transcription, it is most likely that the molecular mechanisms modulating MyoD binding to chromatin are even more various, an issue that awaits further investigation in relation to both physiological and pathological myogenesis.

PARP1 is the most abundant and the best studied family member of the Poly(ADP-ribose) polymerases (PARPs)^25,26^, also termed ADP-ribosyltransferases with diphtheria toxin homology (ARTDs), according to a new nomenclature^27^. PARPs catalyze the addition of single or multiple ADP-ribose units on target proteins, using NAD^+^ as a substrate, leading to Mono(ADP-ribosyl)ation or Poly(ADP-ribosyl)ation (PARylation)^26^. The addition of poly(ADP-ribose) (PAR) polymers is a reversible post-translational modification involved in a variety of cellular processes^28,29^. PARP1 is localized predominantly in the nucleus^28^ and, in part, in mitochondria^30^ and catalyzes the PARylation of many different types of proteins, among which PARP1 itself, histones, transcription factors and other chromatin proteins^28,31^.

The best recognized role of PARP1 is related to the maintenance of genome stability and relies on the modification and recruitment of DNA repair complexes at sites of damaged DNA within chromatin^32^. However, there is increasing evidence that PARP1 influences chromatin dynamics and transcription also in response to a variety of signals other than genotoxic stress, such as inflammation, proliferation and differentiation stimuli^26,33,34^. PARP1 has been reported to influence transcription through a variety of molecular mechanisms, with different outcomes on gene expression. It has long been recognized that PARP1 can directly affect the degree of chromatin compaction. The active enzyme induces chromatin decondensation by causing nucleosomal-histone PARylation^35,36^ and by displacing the linker histone H1 from chromatin^37,38^. On the other hand, inactive PARP1 has been found to function as a structural component of chromatin and to cause chromatin compaction accompanied by transcriptional repression^39–41^.

PARP1 can also indirectly affect the chromatin structure by modulating the pattern of histone modifications and the DNA methylation status^42,43^. For example, active PARP1 promotes histone acetylation at specific promoters^34,44^ and supports histone phosphoacetylation at immediate early response genes during the emergency from quiescence^45^. An additional strategy by which active PARP1 promotes chromatin accessibility, involves the inhibition of EZH2 activity, through PARylation of the histone methyltransferase, and the consequent decrease of the global levels of H3K27me3^46,47^. Moreover, PARP1-dependent PARylation also inhibits the histone demethylase KDM5B, resulting in the increased trimethylation of lysine 4 on histone H3 (H3K4me3)^48^, an active chromatin mark. On the other hand, inactive PARP1 was recognized to be a critical component of the corepressor complexes Groucho/TLE and CtBP, which block transcription from the *MASH1* and *p21* promoters respectively^49,50^. According to a simple and elegant model, PARP1 would play a dual role in the transcriptional regulation, involving its participation in gene repression, when inactive, and then in gene de-repression, following the enzyme activation. In other words PARP-1 would function as an exchange factor promoting the switch from repression to activation in response to particular signals^39,49,50^. Some findings, however, do not fit into this simple model. For example, active PARP1 participates in the establishment and maintenance of heterochromatin during X chromosome inactivation and ribosomal DNA silencing, as well as at telomeres and centromeres^51^. Conversely, inactive PARP1 positively regulates a pluripotency gene expression program in embryonic stem cells^52^.

To complicate the picture, PARP1 functions as a co-regulator of a number of sequence-specific transcription factors, acting in a multifaceted manner. Upon PARylation, some factors, such as YY1, p53, CREB, Sp1, SRY and C/EBPβ, lose the ability to bind to their recognition sequences^53,54^. Some others, such as PPARγ, are instead activated^55^. As a further strategy of co-regulation, PARP1, independently of its catalytic activity, interacts with and co-activates E2F1^56^ and NFkB^57^.

As expected for such a pleiotropic regulator of transcription, it is emerging that PARP1 plays an important role in some developmental and differentiation processes^43,58^. This function was initially underestimated, due to the absence of evident phenotypes, apart from the hypersensitivity to DNA damage, in PARP1 knock-out mice, probably as a consequence of redundant and/or compensatory roles of other PARP family members^59^. Further investigations, based on cellular models of differentiation, demonstrated that PARP1 regulates gene expression at least during adipogenesis^60^, osteoclastogenesis^61^ and neuronal differentiation^49^, as well as during induced pluripotency^52,62^. Despite the large amount of evidence that PARP1 modulates different epigenetic events, most of which also play critical roles in MyoD-dependent transcription, little, if any, is known about the roles of PARP1 in muscle cells, apart from the impact of PARP activity on the cellular bioenergetics of differentiated myotubes^63,64^.

In the present work we provide evidence that PARP1 participates in the transcriptional control of some paradigmatic examples of MyoD target genes by interfering, independently of its catalytic activity, with the recruitment of this myogenic factor to their regulatory regions within chromatin.

## Results

### PARP1 represses MyoD-target gene expression during muscle differentiation

**Figure 1:**
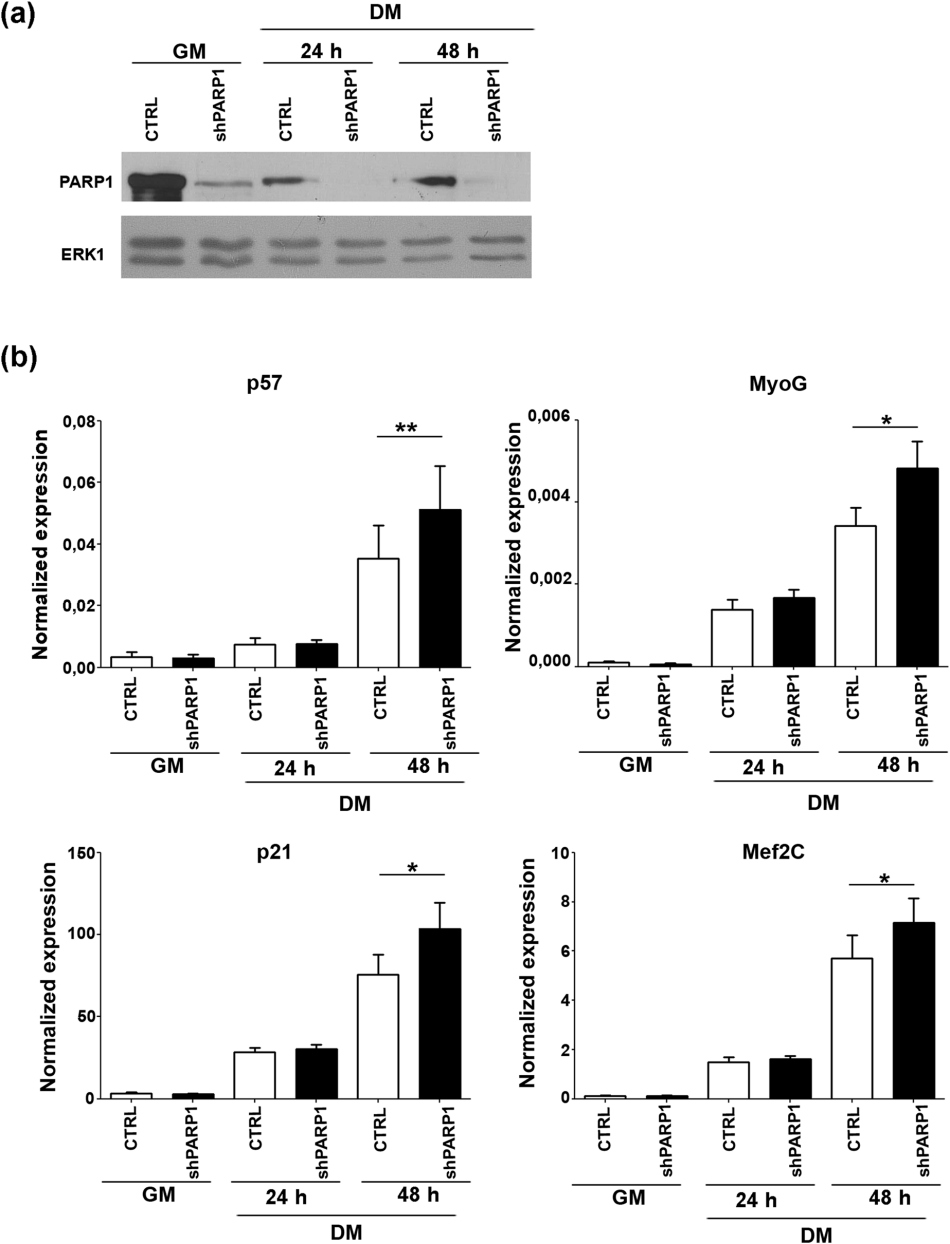
PARP1 depletion boosts the induction of MyoD target genes during differentiation. **(a)** Western blot analysis of PARP1 expression in C2.7 cell pools stably transduced with shPARP1 or with the empty vector (CTRL). Proteins were extracted from cells proliferating in growth medium (GM) or kept for 24 and 48 h in differentiation medium (DM 24 h and DM 48 h respectively. ERK1 was used as an invariant control. One representative experiment of two independent ones is shown. Full length blots are presented in Fig. S8. **(b)** RT-qPCR analysis of *p57*, *myogenin* (MyoG), *p21* and *Mef2C* expression in cells cultured as described in (a). Expression levels, relative to those of Tbp RNA, are reported as the mean ± SEM of six independent experiments. Statistical significance: p-value < 0.05 (*) and < 0.01 (**)**.**

It has been previously reported that PARP1 expression is down-regulated during muscle differentiation, allowing differentiated myotubes to acquire resistance to oxidative stress^63^. According to this observation, and as shown in Supplementary Fig. S1, we found that PARP1 protein levels significantly decrease during the differentiation of C2.7 muscle cells, a well-established and reliable in vitro model of myogenesis, in concomitance with the induction of *p57* and *myogenin*, two examples of direct MyoD targets. This observation hinted at a possible involvement of PARP1 in the control of the muscle differentiation process. To explore this hypothesis, we first examined the effects of PARP1 depletion on muscle gene expression in C2.7 cells stably knocked-down for PARP1. For this purpose, we employed a short hairpin RNA for PARP1 (shPARP1) cloned into a retroviral vector as previously described^45^. Cell pools stably transduced with the shPARP1 or the empty control vector were isolated by puromycin selection and tested for the knockdown efficiency. As reported in Fig. 1a, shPARP1 cells displayed markedly reduced levels of the protein in proliferation conditions and almost undetectable levels in differentiation conditions, compared to control cells. Remarkably, as reported in Fig. 1b, the knock-down of PARP1 correlated with an increased induction of *p57* and *myogenin* expression in differentiated cells. A similar effect was observed on the induction of *p21*, another Cip/Kip inhibitor, and of *Mef2C*, another muscle-regulatory factor, both directly up-regulated by MyoD at early stages of muscle differentiation^1^. The observation that PARP1 depletion did not affect the basal, but only the induced expression of these genes, is easily explained by the well-established occurrence of multiple repressive mechanisms that prevent MyoD activity in proliferating myoblasts^65^ and suggests that PARP1 participates in keeping under control the MyoD-target genes during the differentiation process.

**Figure 2:**
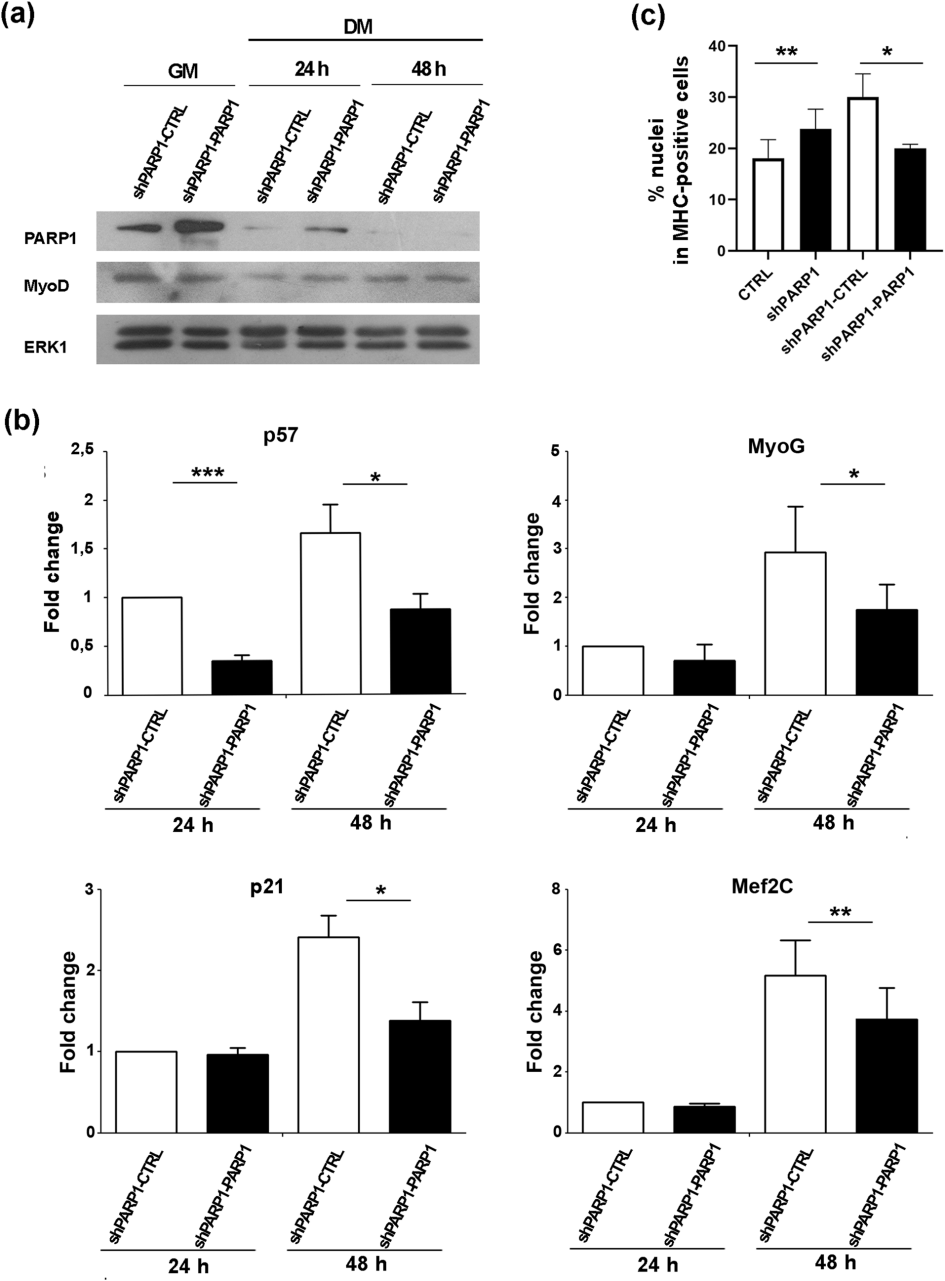
PARP1 re-expression reduces the induction of MyoD target genes during differentiation. **(a)** Western blot analysis of PARP1 and MyoD levels in shPARP1 cells stably transfected with the PARP1 expression vector (shPARP1-PARP1), compared to shPARP1 control cells transfected with the empty vector (shPARP1-CTRL). Proteins were extracted from cells proliferating in growth medium (GM) or kept for 24 and 48 h in differentiation medium (DM 24 h and DM 48 h respectively). ERK1 was used as an invariant control. One representative experiment of two independent ones is shown. Full length blots are presented in Fig. S9. **(b)** RT-qPCR analysis of *p57*, *myogenin* (MyoG), *p21* and *Mef2C* induction in shPARP1-PARP1 compared to shPARP1-CTRL cells. RNA levels were analysed 24 and 48 h after the shift to differentiation medium. Expression levels, relative to those of Tbp RNA and expressed as fold change respect to the control at 24 h, are reported as the mean ± SEM of three independent experiments. Statistical significance: p-value < 0.05 (*)**,** < 0.01 (**), < 0.001 (***)**. (c)** Quantification of the immunofluorescence analysis of MHC-positive myotubes in CTRL, shPARP1, shPARP1-CTRL and shPARP1-PARP1 cells. The percentage of nuclei in MHC-positive cells was calculated as the ratio of the nuclei contained in MHC-positive cells to total number of nuclei, 72 h after shift to the differentiation medium. Representative images are presented in Fig. S3.

To obtain a complementary evidence in support of a repressive role for PARP1 in muscle gene regulation, we analysed the effects of re-expressing the enzyme in PARP1-depleted myoblasts. To this end, we used a construct coding for human PARP1, taking advantage of the significant homology between human and mouse orthologs (about 92% of homology in the protein sequence and 87% in nucleotide sequence). This feature allows the correct function of the human protein in mouse cells and, at the same time, reduces the efficiency by which the short hairpin targets the exogenous PARP1 RNA. shPARP1 myoblasts were co-transfected with either the puc19—human PARP1 vector or with the empty vector, together with a selection vector containing the hygromycin resistance gene. Stable cell pools were selected by hygromycin treatment and first checked for the re-establishment of PARP1 expression. As reported in Fig. 2a, PARP1 expression was restored, at levels comparable to the endogenous PARP1 of control C2.7 cells (Fig.S2). Moreover MyoD levels, which are normally invariant during differentiation^65^, were not affected at all by PARP1 (Fig. 2a). Remarkably, as shown in Fig. 2b, and conversely to the effect of PARP1 depletion, PARP1 re-expression impaired the induction of *p57*, *myogenin*, *p21* and *Mef2C* RNA levels, compared to shPARP1 control cells. It is interesting to note that the repression of *p57*, but not of the other genes, was clearly appreciable as early as 24 h after the shift to differentiation medium, indicating that this gene is more readily sensitive than the others to exogenous PARP1 expression. According to a well established model in which later myogenic markers require both MyoD and some of the early MyoD targets^66^, PARP1 also affected the cell fusion into Myosin Heavy Chain (MHC)–positive myotubes, which is a later event of differentiation. This effect was observed both in depletion and in re-expression experiments (Fig. 2c and Supplementary Fig. S3). The impaired induction of MyoD targets was also observed in response to the transient over-expression of PARP1, as reported for *p57* and *myogenin* in Supplementary Fig. S4.

### PARP1 interacts with the regulatory regions of p57 and myogenin

**Figure 3:**
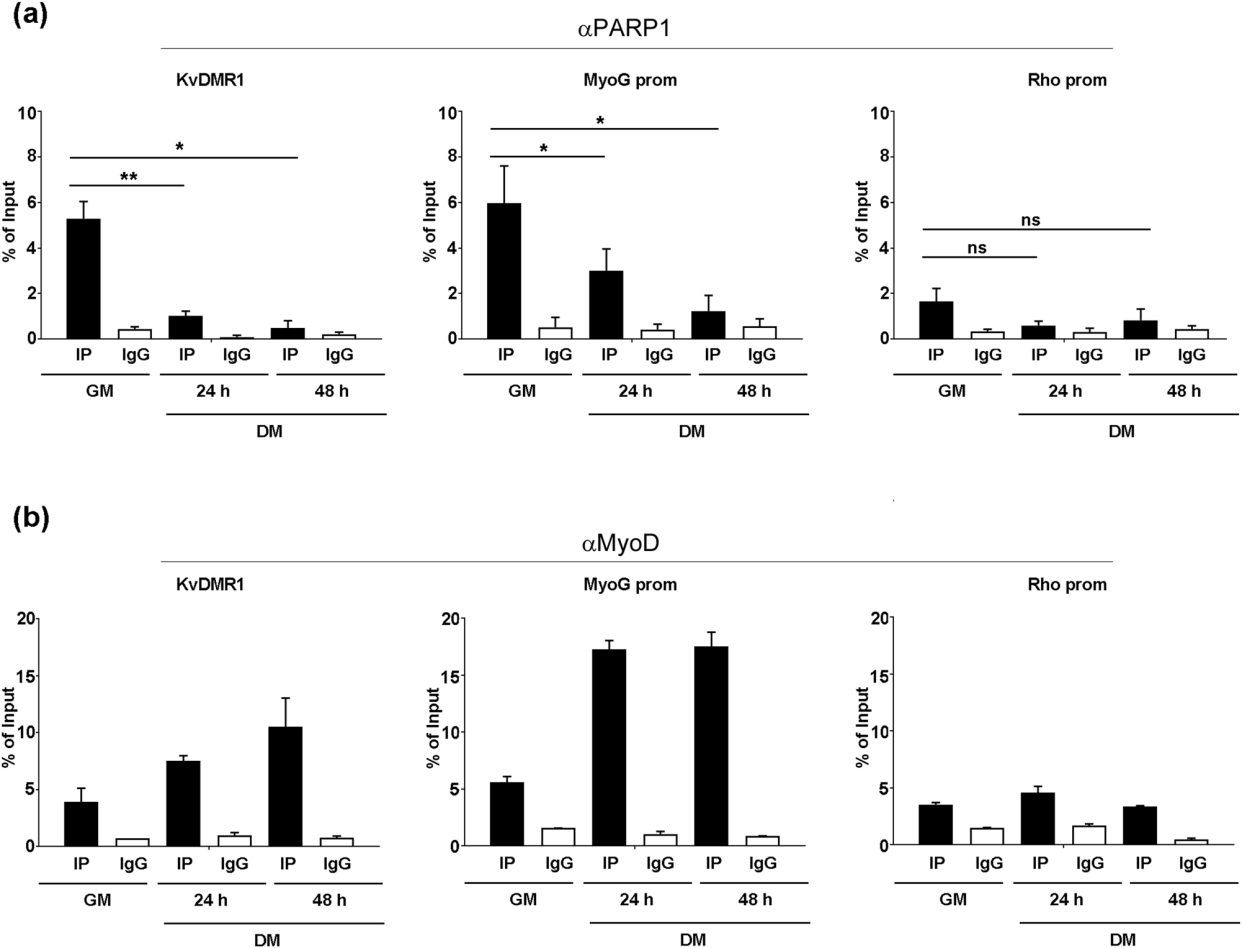
PARP1 interacts with the regulatory regions of the MyoD target genes *p57* and *myogenin*. ChIP-qPCR analysis for PARP1 **(a)** and MyoD **(b)** binding to KvDMR1, *myogenin* promoter (MyoG prom) and *Rhodopsin* promoter (Rho prom), used as a negative control, during C2.7 cell differentiation. Samples were prepared from undifferentiated cells proliferating in growth medium (GM), and from differentiated cells kept for 24 or 48 h in differentiation medium (DM 24 h and DM 48 h respectively) and immunoprecipitated either with the protein specific antibodies (IP samples) or with control IgG. Protein binding to the indicated regions is expressed as percentage of input. The mean ± SEM of four independent experiments is reported for PARP1 ChIP. Statistical significance: p-value < 0.05 (*) and < 0.01 (**). A representative experiment for MyoD ChIP is shown, with error bars corresponding to the mean of each sample analyzed in triplicate.

To explore the molecular mechanism by which PARP1 represses gene expression in differentiating muscle cells, we performed a chromatin immunoprecipitation (ChIP) analysis of PARP1 interaction with chromatin during differentiation. We focused our attention on the *p57* distal regulatory region KVDMR1 and on *myogenin* promoter, critical and well-characterized elements for the differentiation-dependent expression of the two genes. As regards KvDMR1, we have previously demonstrated that this region, unlike *p57* promoter, is directly bound by MyoD upon differentiation and that its epigenetic status influences the recruitment of the myogenic factor and the consequent disruption of a repressive chromatin loop with *p57* promoter^15,17–19^. Concerning *myogenin* promoter, this region has always been a paradigmatic model for the study of MyoD-induced transcription and has been characterized in great detail for the dynamics of histone modifications and transcription factor binding during differentiation^67^. Nuclear extracts were collected from undifferentiated and differentiated cells and analysed for the enrichment of KvDMR1 and *myogenin* promoter in PARP1 immunoprecipitates. *Rhodopsin* (Rho) promoter was used as a negative control, as previously reported^45^. As shown in Fig. 3, PARP1 binds to both KVDMR1 and *myogenin* promoter, but not to Rho promoter, suggesting its involvement in the regulation of *p57* and *myogenin* transcription. Moreover, in line with the decrease of the total protein levels observed during differentiation, the association of PARP1 with both regions was reduced in differentiated respect to undifferentiated cells, supporting the hypothesis that the binding of the enzyme to the regulatory regions of *p57* and *myogenin* might inhibit their induction. A ChIP assay for MyoD binding to the same regions confirmed our previous findings and indicated the recicprocal occupancy of these two proteins. Moreover, as reported in Supplementary Fig. S5, in undifferentiated cells PARP1 also binds to p57 promoter and to an intragenic p57 regulatory region, which we have previously described to be regulated by MyoD, the first through and indirect mechanism^19^ and the second through direct MyoD binding^15^.

### The presence of PARP1 interferes with MyoD binding to KvDMR1 and myogenin promoter

**Figure 4:**
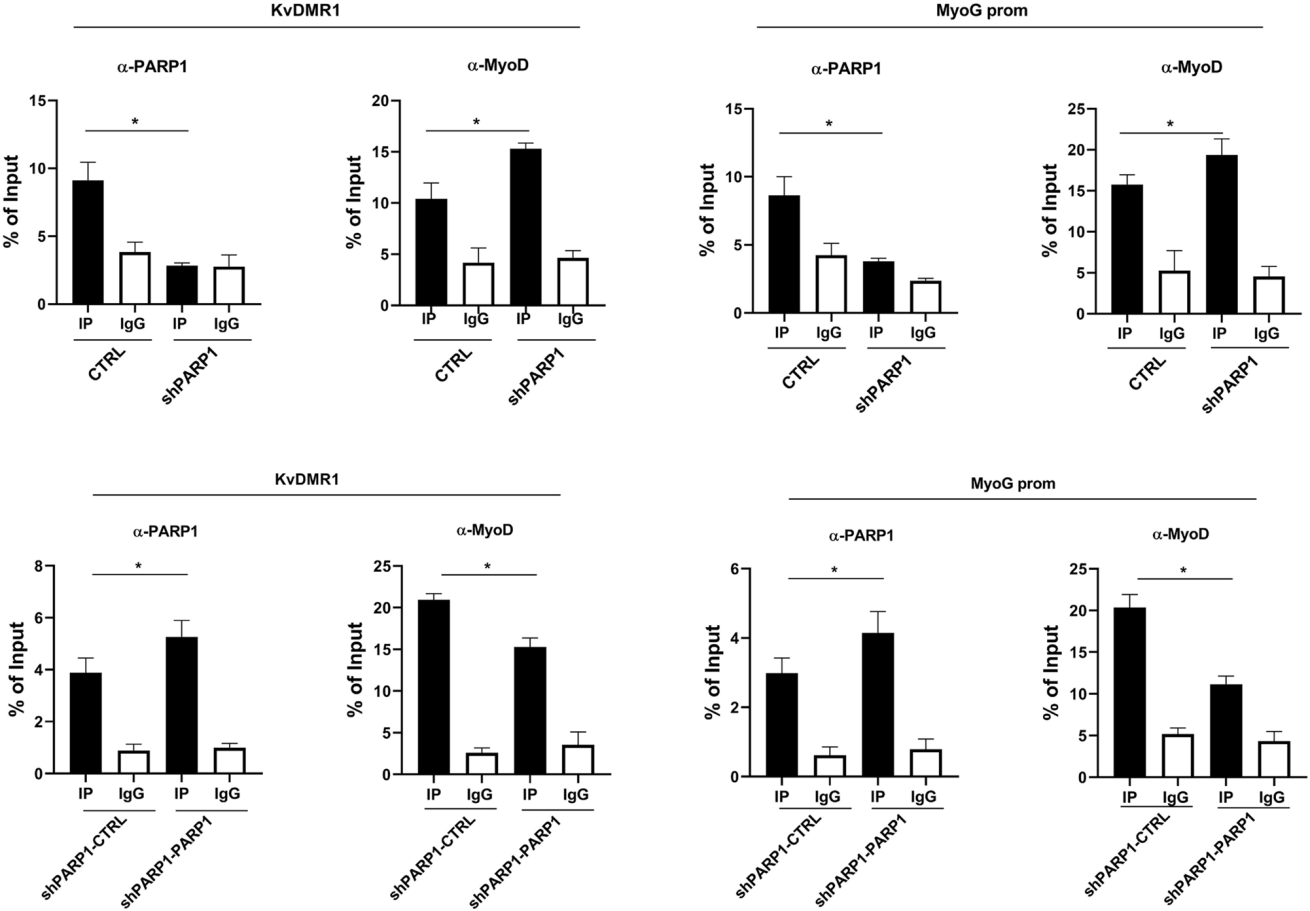
PARP1 impairs MyoD binding to *p57* and *myogenin* regulatory regions. ChIP-qPCR analysis for PARP1 and MyoD binding to KvDMR1 and *myogenin* promoter (MyoG prom). Samples were prepared from CTRL, shPARP1, shPARP1-CTRL and shPARP1-PARP1 cells, collected 24 h after the shift to differentiation medium, and then immunoprecipitated with PARP1 or MyoD antibodies (αPARP1 and αMyoD, IP samples), as well as with control IgG. Protein binding is expressed as percentage of input and represents the mean ± SEM of three independent experiments. Statistical significance: p-value < 0.05 (*).

As mentioned above, the impairment of MyoD recruitment to its binding elements, due to chromatin restraints, is involved in the failure of MyoD-dependent gene activation in undifferentiated cells or in cells refractory to express some specific MyoD target genes. Considering that the association of PARP1 with KvDMR1 and *myogenin* promoter declines during differentiation, in concomitance with the onset of MyoD recruitment, we speculated that PARP1 could repress *p57* and *myogenin* expression by interfering with the binding of MyoD to their regulatory regions. To verify this hypothesis, we evaluated the relationship between PARP1 and MyoD association with KvDMR1 and *myogenin* promoter in cells silenced for PARP1 and in cells re-expressing PARP1 compared to their respective controls. ChIP assays for the interaction of PARP1 or MyoD with KvDMR1 and *myogenin* promoter were performed in cells cultured in differentiation conditions, in which MyoD is normally recruited to these regions. As shown in Fig. 4, cells depleted of PARP1 show decreased PARP1 binding, as expected, while cells re-expressing PARP1 showed increased PARP1 binding to both regions. Remarkably, as shown in the same figure, the PARP1 recruitment inversely correlated with that of MyoD, suggesting that PARP1 may act by hindering the MyoD binding to its targets.

### PARP1 activity is not required for p57 and myogenin repression

**Figure 5:**
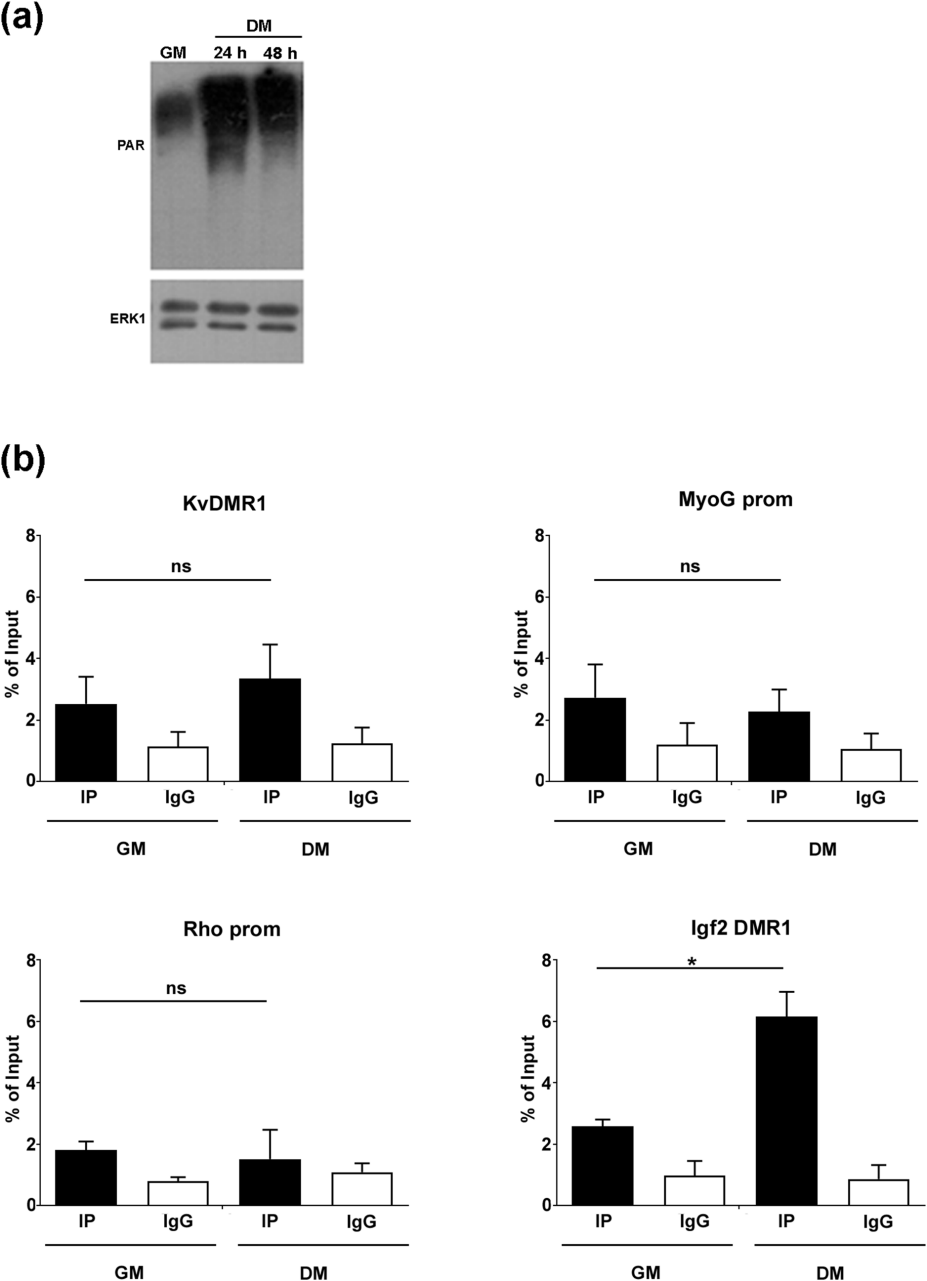
PAR levels at the regulatory regions of the MyoD target genes *p57* and *myogenin* do not change during differentiation. **(a)** Western blot analysis of PARylated proteins in undifferentiated C2.7 myoblasts proliferating in growth medium (GM) and in differentiated C2.7 myotubes cultured for 24 or 48 h in differentiation medium (DM). PARylated proteins were detected with the anti-PAR antibody. ERK1 was used as an invariant control. Full length blots are presented in Fig. S10. **(b**) ChIP-qPCR analysis for PAR levels at KvDMR1, *myogenin* promoter (MyoG prom), *Rhodopsin* promoter (Rho prom), and Igf2 Differentially Methylated region (Igf2 DMR1) during C2.7 cell differentiation. Samples were prepared from undifferentiated cells proliferating in growth medium (GM), and from differentiated cells kept for 24 h in differentiation medium (DM) and immunoprecipitated either with PAR antibody (IP samples) or with control IgG. Protein binding is expressed as percentage of input and represents the mean ± SEM of four independent experiments for KvDMR1 and MyoG prom and of three independent experiments for Rho prom and Igf2 DMR1. Statistical significance: p-value < 0.05 (*); ns stands for not significant.

To investigate the possible role of the enzyme activity in the observed effects, we first measured the total PAR levels during differentiation. Unexpectedly, as demonstrated by the western blot analysis reported in Fig. 5a, PARylation strongly increased upon the induction of differentiation, at the opposite of PARP1 levels. A comparable result was obtained using a different protocol for cell lysis (Supplementary Fig. S6). Several hypotheses can be advanced to explain the observed increase (see “Discussion” Section). However, to address more directly whether PARylation was involved in the regulation of MyoD targets, we evaluated the dynamics of PAR accumulation on the *p57* and *myogenin* regulatory regions during differentiation. In light of the technical difficulties reported for the study of chromatin PARylation and, in particular, considering that cell lysis can induce DNA damage and artificial PARylation of non-specific chromatin targets ^68^, we also analysed two additional regulatory regions, as negative and positive controls respectively. One is Rho promoter, that we have previously shown to be scarcely PARylated ^45^. The other one is Igf2 DMR1 (a differentially methylated region located upstream of the *Igf2* promoter 1), which was previously reported to be susceptible to PARylation ^69^. As reported in Fig. 5b, while PARylation was constantly low at Rho promoter, it increased at Igf2 DMR1 during differentiation, reflecting at least in part the trend of the global PARylation observed in cell extracts. In contrast, the PAR levels at p57 and myogenin regulatory regions, which were only a little higher respect to Rho promoter, did not change upon differentiation. The lack of correlation between the transcriptional activity of the two genes and the PAR levels at KVDMR1 and *myogenin* promoter represented a first indication that PARylation did not play a role in their regulation.

**Figure 6:**
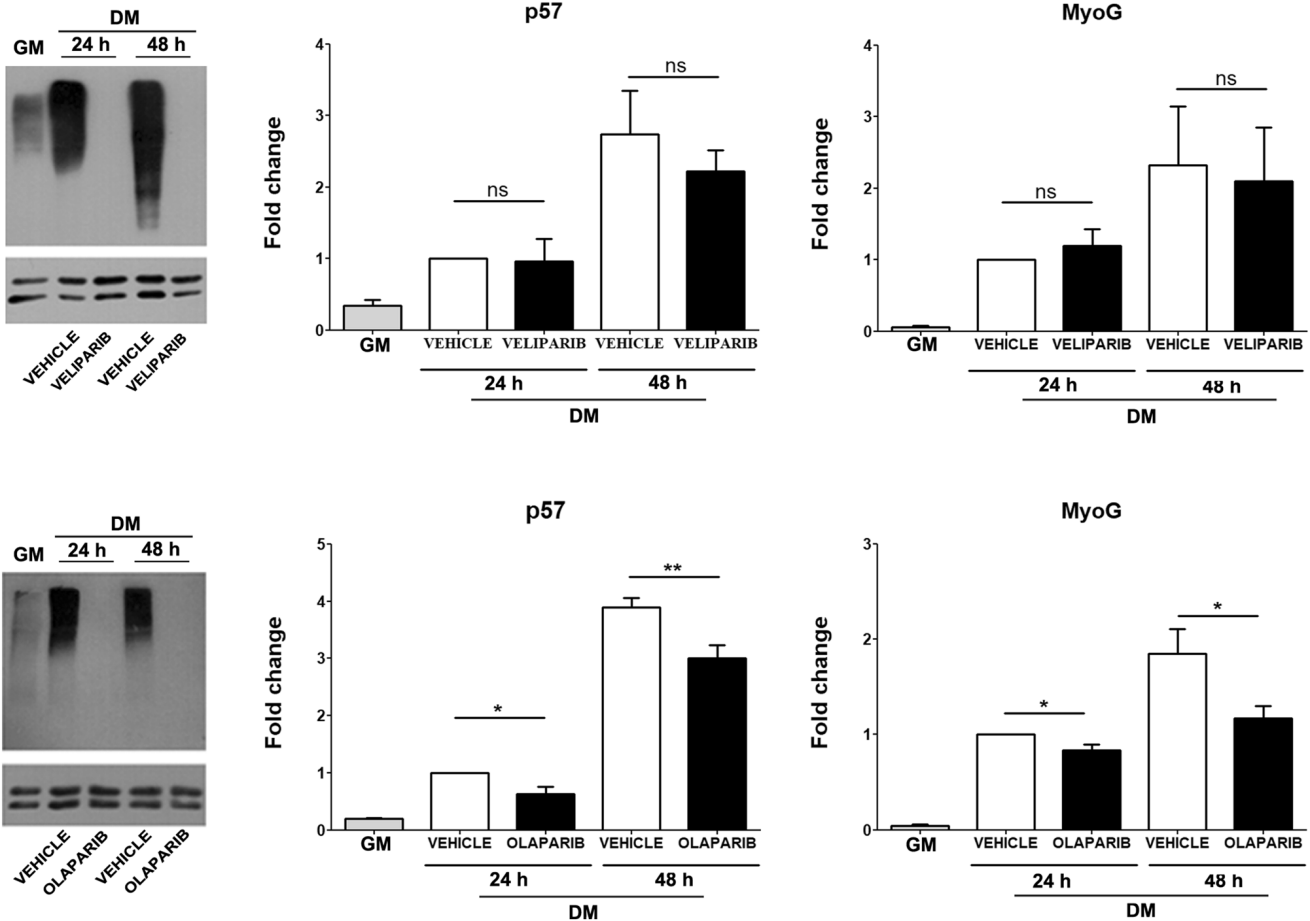
PARP activity is not required for the repression of *p57* and *myogenin* induction. Left panels: western blot analyses of PAR levels in C2.7 differentiating in the presence of either Veliparib (upper) or Olaparib (lower). Protein samples were prepared from cells proliferating in growth medium (GM) and from cells shifted to differentiation in medium (DM), in the presence of 1 µM Veliparib, 1 µM Olaparib or the control vehicle DMSO (vehicle) and kept for 24 and 48 h. PARylated proteins were detected with the anti-PAR antibody. ERK1 was used as an invariant control. One representative of three independent experiments is shown. Full length blots are presented in Fig. S11. Right panels: RT-qPCR analysis of *p57* and *myogenin* (MyoG) expression in C2.7 cells differentiated in the presence of Veliparib (upper right) or Olaparib (lower right), as described for western blot analyses. Expression levels, relative to those of Tbp RNA and expressed as fold change respect to the control at 24 h represent the mean ± SEM of three independent experiments. Statistical significance: p-value < 0.05 (*) and < 0.01 (**); ns stands for not significant.

To directly address whether the transcriptional control of *p57* and *myogenin* by PARP1 was independent of the enzyme activity, we measured the expression of the two genes in myoblast cells induced to differentiate in the presence of pharmacological PARP inhibitors. To this end we used the small molecules ABT-888 (Veliparib) and AZD-2281 (Olaparib) two PARP inhibitors selective for PARP1 and PARP2 family members^70^ and capable of strongly inhibiting PARylation at concentrations that do not affect cell viability^70,71^. As expected, and as reported in Fig. 6 (left panels), either Veliparib or Olaparib treatment effectively abrogated global PAR levels in differentiating cells. However, the inhibition of the enzyme activity, unlike the depletion of the protein, did not promote, rather it slightly inhibited *p57* and *myogenin* induction (Fig. 6), indicating that PARP1 represses the expression of the two genes through a catalytic-independent mechanism.

**Figure 7:**
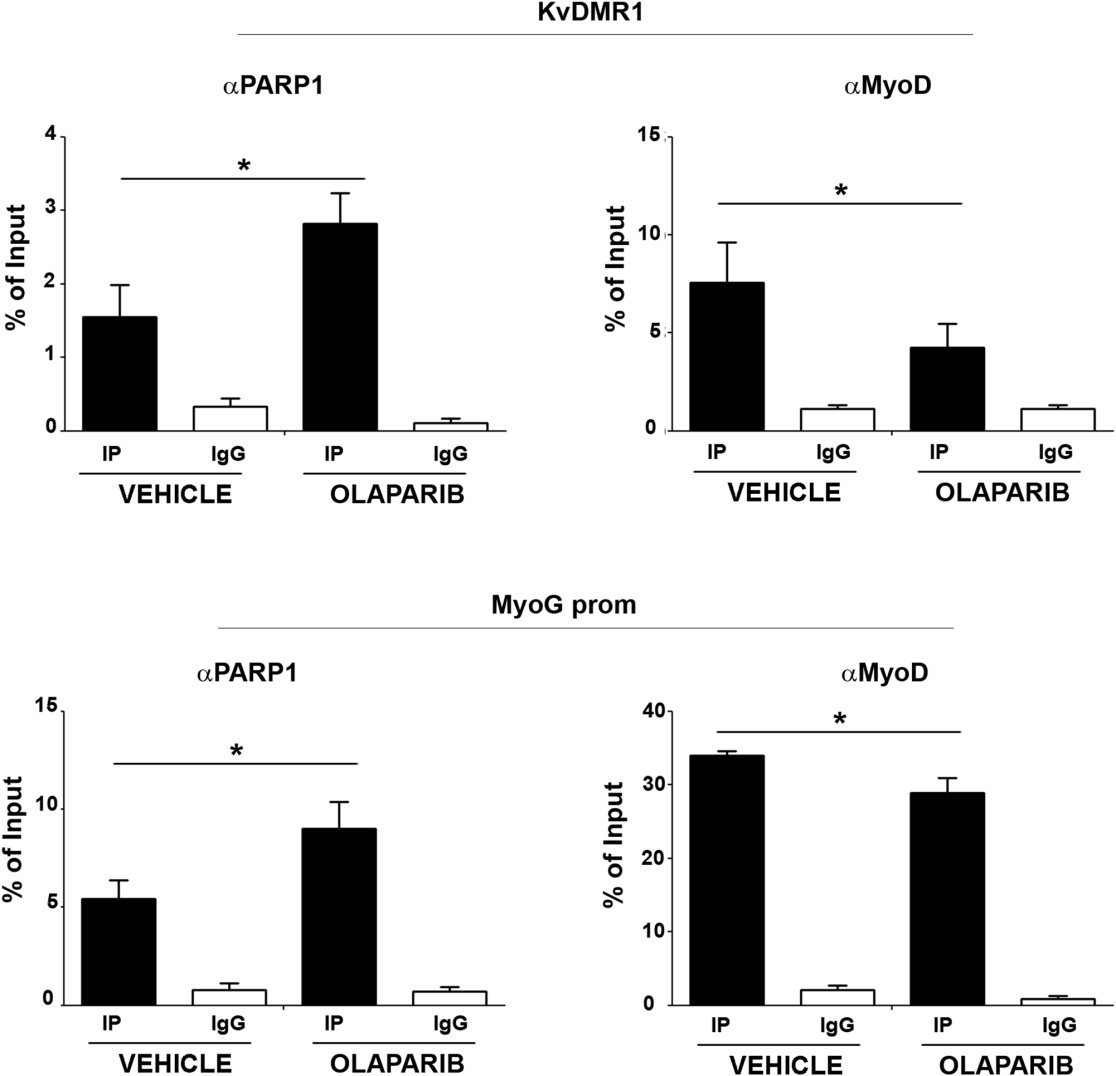
Olaparib treatment results in increased PARP1 binding and decreased MyoD recruitment to p57 and myogenin regulatory regions. ChIP-qPCR analysis for PARP1 and MyoD binding to KvDMR1 and *myogenin* promoter (MyoG prom). Samples were prepared from C2.7 cells collected 24 h after the shift to differentiation medium in the presence of either 1 µM Olaparib or the control vehicle DMSO (VEHICLE), and then immunoprecipitated as described in Fig. 4. Protein binding is expressed as percentage of input and represents the mean ± SEM of three independent experiments. Statistical significance: p-value < 0.05 (*).

Interestingly, we noticed that the effect of Olaparib was more evident respect to that of Veliparib and also statistically significant. It is worth mentioning that Olaparib was reported not only to strongly inhibit PARP1 activity, but also to cause the trapping of PARP1 on the chromatin^71,72^. This effect, ascribed both to the inhibition of the enzyme activity and to the induction of a conformational change of PARP1^72^, was especially detected in the presence of DNA strand breaks. However, a slight, but detectable increase of PARP1 binding to chromatin was also observed in cells treated with Olaparib in the absence of DNA damaging agents^71^. To verify whether Olaparib treatment affected PARP1 binding to MyoD targets, we performed ChIP assays in C2 cells differentiated in the presence or absence of Olaparib treatment. Interestingly, as reported in Fig. 7, Olaparib treatment actually resulted in increased PARP1 binding to both KvDMR1 and *myogenin* promoter in differentiated cells. The molecular mechanisms of PARP1 trapping by PARP inhibitors have not been completely clarified yet^73^, nor we can say whether the increased PARP1 binding to muscle genes after Olabarib treatment involves its trapping on DNA breaks. This issue will certain deserve further investigation. However, and remarkably, increased PARP1 binding correlated with decreased MyoD recruitment to p57 and myogenin regulatory regions (Fig. 7), similarly to what observed when PARP1 was re-expressed in shPARP1 cells (Fig. 4) and in line with the impaired induction of the two genes (Fig. 6). These findings not only substantiated the inhibitory role of PARP1 in MyoD binding to chromatin, but also confirmed that this function does not require the enzyme activity or even that it is reinforced by its inactivity.

### PARP1 impairs the accumulation of H3K4me3 at MyoD binding regions

**Figure 8:**
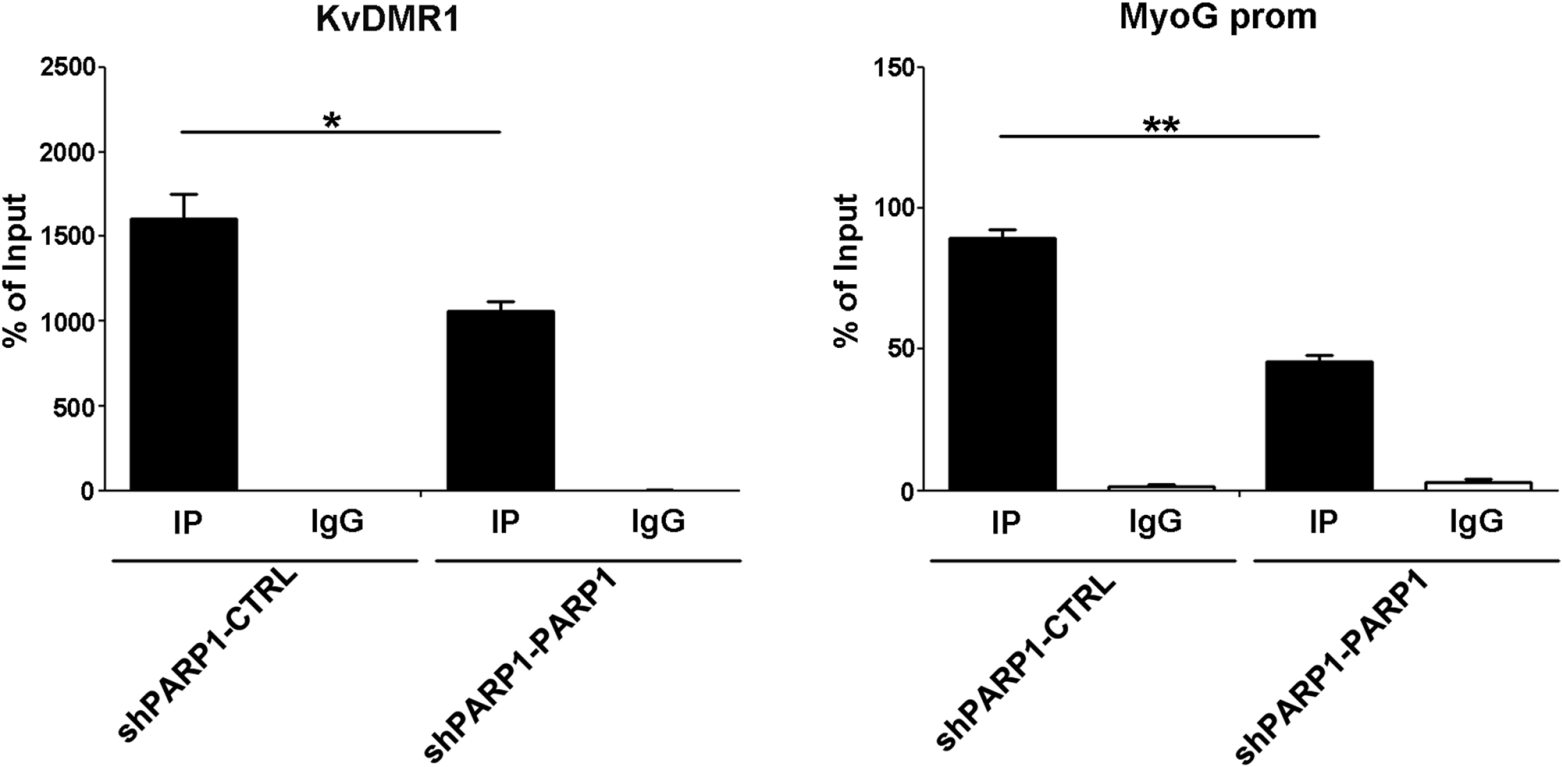
PARP1 re-expression impairs H3K4me3 accumulation at *p57* and *myogenin* regulatory regions. ChIP-qPCR analysis for H3K4me3 association to KvDMR1 and *myogenin* promoter. shPARP1 cells stably re-expressing PARP1 (PARP1) and from shPARP1 control cells (CTRL) were collected 24 h after the shift to differentiation medium and samples were immunoprecipitated either with the H3K4me3 antibody (IP samples) or with the control IgG. Values are expressed as percentages of input and represent the mean ± SEM of three independent experiments. Statistical significance: p-value < 0.05 (*) and < 0.01 (**).

**Figure 9:**
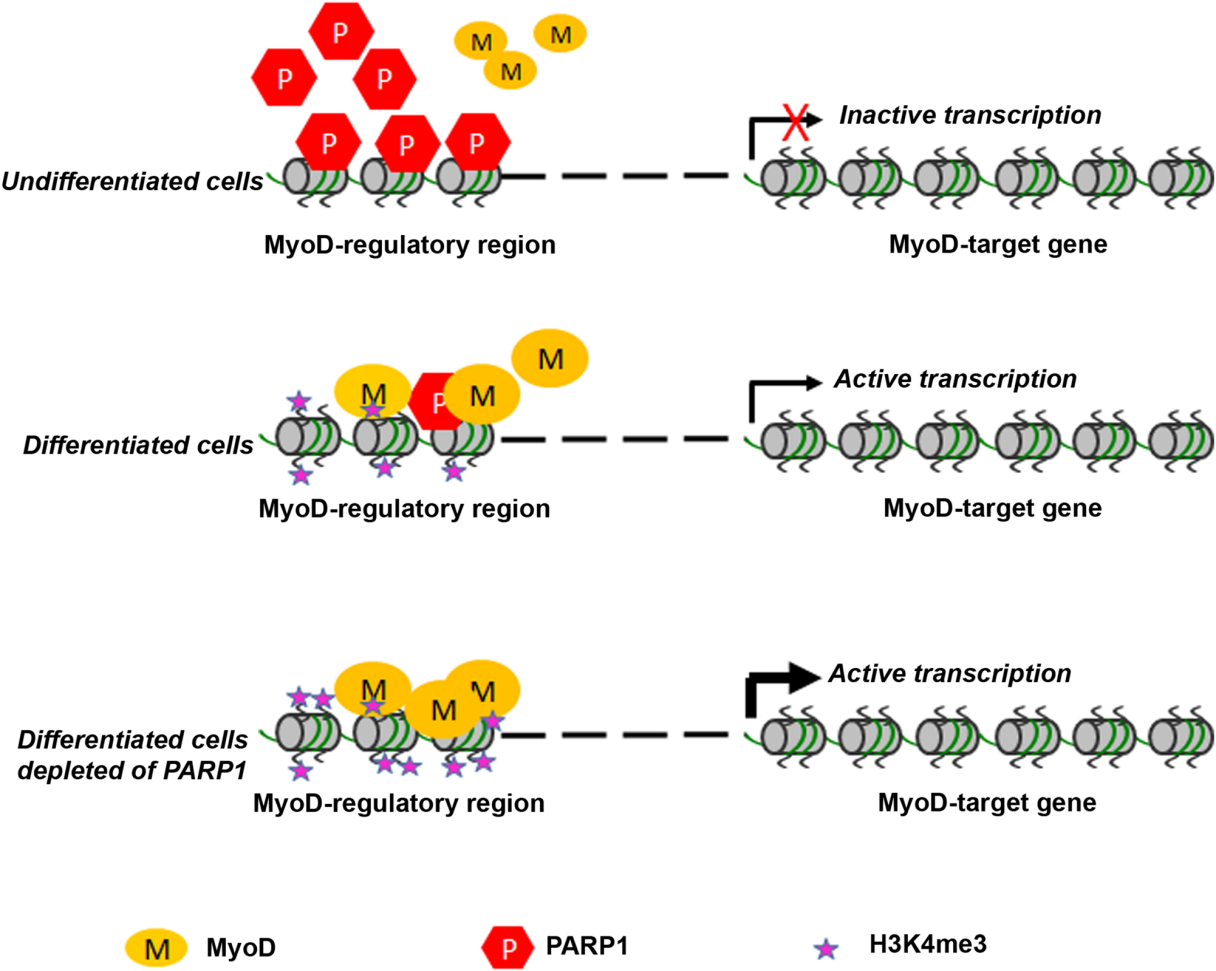
A hypothetical model for the functional interference of PARP1 with MyoD-dependent transcription. In undifferentiated cells the absence of MyoD activity at the regulatory regions of its targets prevents the induction of muscle genes, concomitantly with the presence of high levels of PARP1. Upon differentiation, the reduction of PARP1 levels and its decreased association with the regulatory regions increases the binding of MyoD and the induction of gene expression, accompanied by the accumulation of H3K4me3. When PARP1 is depleted, the binding of MyoD and the accumulation of H3K4me3 to the regulatory regions are further facilitated, resulting in increased up-regulation of MyoD targets.

Although it cannot be excluded that PARP1 acts by directly hindering MyoD recruitment to its binding sequences, it is reasonable to hypothesize that PARP1 may act by maintaining a repressive chromatin environment at genes that have to be induced at the onset of differentiation. PARP1 affects chromatin function through multiple strategies, including the alteration of some types of histone modifications, among which the tri-methylation of lysine 4 in histone H3 (H3K4me3), an active chromatin mark. PARP1 was reported to affect H3K4me3 in a complex manner. In particular, in its active state, the enzyme inhibits the H3K4me3 histone demethylase KDM5B, promoting the accumulation of this modification on multiple gene promoters^48^. On the other hand, inactive PARP1 inhibits the H3K4 methyltransferase MLL1, and prevents H3K4me3 accumulation at the gene promoter of interleukin 6, repressing its expression^74^. By inspecting the ChIP-seq data for muscle cells from ENCODE/Caltech, we noticed that the levels of H3K4me3 at KvDMR1 and *myogenin* promoter, were lower in undifferentiated respect to differentiated cells. To assess the possible relationship of these chromatin changes with the differential binding of PARP1, we performed ChIP assays for H3K4me3 in cells re-expressing PARP1 compared to silenced cells. Remarkably, as shown in Fig. 8, the accumulation of H3K4me3 at KvDMR1 and *myogenin* promoter was reduced upon the re-expression of PARP1, presumably as a result of its increased binding to chromatin in differentiating cells (as shown above in Fig. 4). In line with this evidence, identical and specular results were obtained when comparing Olaparib-treated with untreated cells and when comparing PARP1-silenced cells with their control, respectively (Supplementary Fig. S7). All these findings suggest the existence of a functional link between the loss of PARP1 binding, the accumulation of the activating histone modification H3K4me3, the recruitment of MyoD and the up-regulation of MyoD-target genes (see the model depicted in Fig. 9).

## Discussion

In the present work we provide evidence, for the first time, that PARP1 participates in the control of gene expression during muscle differentiation and, in particular, we show that PARP1, independently of its enzymatic activity, functions as negative regulator of MyoD binding to chromatin. These findings not only add new insight into the transcriptional control of muscle differentiation, but also expand our knowledge about the differentiation-related functions of PARP1.

We found that PARP1 depletion promotes the induction of *p57*, *myogenin*, *Mef2C* and *p21*, while PARP1 re-expression reduces their up-regulation. The small, yet statistically significant changes in gene expression, observed when PARP1 levels are modulated, are accompanied by similarly small changes in later events of differentiation, such as the fusion into myotubes. However, it is conceivable that a refined modulation of muscle gene expression may play a critical role during in vivo processes of myogenesis, such as muscle development or regeneration, which take place over more extended time periods respect to in vitro differentiation.

Importantly, our finding that PARP1 affects at least four different genes, which are known to be induced with different kinetics and through different molecular mechanisms, but which share the property to be directly targeted by MyoD, strongly supports a functional interference of PARP1 with MyoD-dependent transcription.

PARP1-dependent gene repression correlates with the presence of the protein on the regulatory regions of the affected genes, as demonstrated by a wide-range analysis of gene expression in MCF7 tumor cells^75^ and in human embryonic stem cells^76^ as well as for specific genes such as *PARP1* itself in Ewing's sarcoma cells^77^ and *IL6* in the RAW 264.7 macrophage cell line ^74^. Accordingly, we found that PARP1 directly interacts both with *myogenin* promoter, the prototypical regulatory region targeted by MyoD^67^, and with KvDMR1, a distant imprinting control region that we identified as a MyoD-responsive element involved in the control of *p57* induction during differentiation^17–19^. Moreover, consistent with the decrease of total PARP1 levels and with the need of relieving a negative control from these genes to allow their up-regulation, PARP1 binding was significantly reduced in differentiated respect to undifferentiated cells. It is worth noticing that the loss of PARP1, although necessary, does not appear sufficient for inducing MyoD targets, as indicated by the observation that the basal expression of these genes, in undifferentiated cells, was not up-regulated upon PARP1 depletion. However, this is not surprising when considering that MyoD, although expressed since before differentiation, is kept inactive by interacting proteins and post-translational modifications induced by proliferation pathways^65^. For this reason, although it is possible that PARP1 participates in the repression of MyoD-targets in undifferentiated cells, the principal suggestion emerging from our data is that the role of PARP1 is to limit the up-regulation of muscle genes during differentiation (see the model depicted in Fig. 9).

Depending on the regulatory context, PARP1 can either promote or suppress transcription and each of these effects can be either dependent or independent of its catalytic activity^78^. In this regard, much more information has been gathered about the catalytic-dependent respect to the catalytic-independent functions of PARP1. In light of the down-regulation of PARP1 expression, we were surprised to see a strong induction of PARylation during differentiation. We cannot say at this moment whether increased PARylation can be ascribed to a strong activation of the small amount of residual PARP1 or to other PARP family members. Concerning the molecular mechanism inducing PARP activity, one possible explanation is the accumulation of DNA strand breaks, which has long been noticed in differentiated myotubes as well as in other types of terminally differentiated cells^79^. Although the possible role of this phenomenon in differentiation processes remains hypothetical, it has been suggested that the accumulation of DNA damage involves a reduced efficiency of DNA repair at global genome level, yet its maintenance at active genes^80^. This phenomenon is certainly interesting and will deserve further investigation. However, in the context of the present work we obtained evidence that the enzyme activity is not required for PARP1 to restrain *p57* and *myogenin* induction. At first, we observed that the levels of PARylation at the regulatory regions of these genes do not correlate with their transcriptional activity. Most importantly, the inhibition of PARP activity by each of two different inhibitors, Veliparib and Olaparib, does not increase *myogenin* and *p57* expression as PARP1 depletion does. It is important to notice that, while Veliparib treatment does not affect their expression, Olaparib treatment even represses their induction. The effect of Olaparib, that we found to correlate with the maintenance of inactive PARP1 at *p57* and *myogenin* regulatory regions, possibly due to its trapping, further reinforced the suggestion that PARP1 represses the expression of these genes by affecting the chromatin function in a catalytic-independent manner.

Regarding the molecular mechanism of repression, we observed that the binding of PARP1 inversely correlates with that of MyoD. This kind of effect is opposite to that observed for other transcription factors, such as NF-KB^81^ and SOX2^52^, whose binding to chromatin is enhanced by inactive PARP1. In light of our previous finding that MyoD functionally interacts with CTCF at KvDMR1, to induce *p57* expression^18^, and considering the existence of an interplay between PARP1 and CTCF in promoting chromatin insulation and in repressing ribosomal transcription^82^ , we wondered if CTCF was involved in the observed effects of PARP1 on MyoD function. However, it is very unlikely that this is the case, since PARP1-dependent regulation of CTCF requires CTCF PARylation^83^, whereas we found that the ability of MyoD to activate its targets is not influenced by the inhibition of PARP activity.

Interestingly, although active PARP1 inhibits the histone demethylase KDM5B through its PARylation, which results in the accumulation of the active mark H3K4me3 at several genomic regions^48^, it has been reported that inactive PARP1 prevents the deposition of the same histone modification at IL-6 promoter, through the inhibition of the histone methyltransferase MLL1, leading to the suppression of *IL-6* expression^74^. In line with this report, we found that the accumulation of PARP1 inversely correlates with that of H3K4me3 at KvDMR1 and *myogenin* promoter. Since MLL1 is highly expressed in muscle cells and seems to regulate both proliferation and differentiation^84^, the possibility of its functional interaction with PARP1 during myogenesis will deserve further investigation. Although it is conceivable that the decreased levels of H3K4me3, by promoting a repressive chromatin environment, are responsible for the PARP1-dependent exclusion of MyoD from its targets, we still cannot exclude that the accumulation of H3K4me3 is a result of transcriptional activation consequent to MyoD binding. In fact, while the presence of H3K4me3 is clearly associated with gene activity, some lines of evidence suggest that this modification does not play an instructive role in transcription but, rather, is a result of this^85^. An alternative hypothesis for explaining the interference of PARP1with MyoD binding could be related to the role of PARP1 as a structural component of the chromatin and to its ability to directly interact with nucleosomes. In fact, in the absence of the enzyme activity and, similarly to histone H1, PARP1 was found to promote the compaction of in vitro-assembled chromatin^39^. Interestingly, it has been recently suggested that inactive PARP1 can enhance the chromatin binding of the pioneer transcription factor SOX2 by distorting nucleosomal DNA^52^. Actually, the effect exerted by PARP1 on MyoD binding is the opposite. However, it is possible to imagine that the ability of PARP1 to alter the structural features of nucleosomal DNA may affect in different ways the binding of different transcription factors. The exact mechanisms by which inactive PARP1 affects the ability of MyoD to bind and transactivate its targets await an in depth investigation aimed at defining in detail the status of the chromatin accessibility, the pattern of additional histone modifications and the structural features of nucleosomes.

In summary, this work reveals that PARP1 affects the ability of MyoD to access at least some of its chromatin targets during differentiation, highlighting a novel player in the epigenetic control of muscle differentiation. In this regard, it would be exciting to be able to generalize the present findings through a comparative analysis of MyoD and PARP1 binding throughout the genome, in relation with chromatin dynamics and gene expression, during the differentiation process. It would also be interesting to examine the possible role of PARP1-dependent chromatin alterations in muscle pathologies, such rhabdomyosarcoma, in which the failure to activate some myogenic targets correlates with the defective MyoD binding to their regulatory regions^24^. It is worth mentioning that the PARP inhibitor Olaparib, already approved for the therapy of some tumor types defective for homologous recombination DNA repair, is currently under investigation for the possible treatment of inflammatory and oxidative stress diseases^86^. In this regard, Olaparib was reported to improve the mitochondrial function in skeletal muscle cells, suggesting the possible clinical use of this inhibitor to treat muscle dysfunctions associated with mitochondrial defects^64^. Therefore, the attainment of an integrated picture of the complex and multiple roles of PARP1 in muscle is extremely important for increasing our awareness of the pleiotropic effects of modulating PARP1 function in different pathophysiological contexts.

## Methods

### Cell cultures

Mouse C2.7 muscle cells were grown in Dulbecco’s modified Eagle’s medium (DMEM) (Gibco) supplemented with 10% fetal bovine serum (FBS) (Microgem) and maintained sub-confluent in order to avoid differentiation. To induce MyoD activity and differentiation, cells were shifted to DMEM supplemented with 0.5% FBS (differentiation medium) and collected 24 or 48 h later, as indicated. 1 µM Olaparib (Selleckchem) or 1 µM Veliparib (Selleckchem) were administered to cell cultures at the time of the shift to differentiation medium.

### Generation of stably knocked- down cell lines and cell transfections

The shPARP1 retroviral vector was previously constructed, as described^45^.

Empty (Control) and shPARP-1-expressing retroviruses were produced by transfecting the constructs into BOSC 23 packaging cells as previously reported^45^. For the stable knock-down of PARP1, C2.7 cells were incubated with the BOSC23 retroviral supernatant supplemented with 4 μg/ml polybrene for 8 h and then re-fed with fresh medium. 48 h after infection cells where seeded at low density in selective medium containing 2.5 μg/ml of puromycin (Sigma) and, after about one week, the surviving clones were pooled together and cultured as indicated for C2.7 cells.

Stable re-expression of PARP1 was obtained by co-transfecting the puc19-PARP1 plasmid, coding for human PARP1 and kindly provided by Prof. Paola Caiafa, with the pSV2hygro plasmid, coding for the hygromycin resistance, in ratio 10:1, into shPARP1 C2.7 cells, using Lipofectamine 2000 reagent (Invitrogen) according to the manufacturer’s instructions. 48 h later, cells transfected with either puc19-PARP1 plasmid or with the empty vector puc19 were seeded in selective medium containing 2.5 μg/ml of hygromycin (Sigma) and, after two weeks, the surviving clones were pooled together and cultured as indicated for C2.7 cells.

### Western Blot assays

Cells were collected in RIPA buffer (NaCl 150 mM, NP-40 1%, Sodiumdeoxycholate 0.5%, SDS 0.1% and TRIS–HCl pH 8.0 50 mM) or in Laemmli 1X buffer (TRIS–HCl ph 6.8 60 mM, SDS 2%, glycerol 10%, β-mercaptoethanol 12.5%, Toluidine Blue 0.1 g). Proteins were resolved by electrophoresis in 8% polyacrylamide SDS-PAGE and transferred to nitrocellulose membranes by electro-blotting. Membranes were blocked in 2% non-fat dry milk in TBS containing 0.05% Tween 20 for 1 h at room temperature and incubated with the primary antibodies overnight at 4 °C, then washed three times and incubated with peroxidase-conjugated anti-mouse or anti-rabbit secondary antibodies (Bio-Rad). Proteins were detected using the ECL chemiluminescence system (Cyanagen).

The following primary antibodies used for western blot were purchased from Santa Cruz Biotechnology: anti-PARP1 (H-250, sc-7150), anti-PAR (10H, sc-56198), anti-p57 (H-91, sc-8298) and anti-ERK1 (C-16, sc-093). The F5D hybridoma supernatant was used for myogenin.

### Gene expression analysis

Total RNA was extracted using the High Pure RNA Isolation Kit according to the manufacturer's instructions (Roche Diagnostics) and 500 ng were reverse transcribed with the iScript cDNA Synthesis Kit (Bio-Rad). Realtime-PCR (qPCR) reactions were performed in 10 μl of reaction buffer containing 1 μl of diluted cDNA in 9 μl of GoTaq qPCR Master Mix (Promega) containing each primer at the optimized final concentration (150-250 nM). The reaction was performed in the termocycler “MiniOpticon Real-Time PCR detection system” (Bio-Rad) or "CFX96 Real-Time PCR detection system" (Bio-Rad). The primer pair efficiency, the normalized expressions (ΔΔCt) and the Standard Error for the Normalized Expression were determined with CFX ManagerTM software (Bio-Rad).

The following primer sets were used:

- for *p57*
  - F: 5′-AACTTCCAGCAGGATGTGCC-3’
  - R: 5′-CATCCACTGCAGACGACCAG-3′;
- for *myogenin*
  - F: 5′-GTCTCTTCCTGAAGCCAGTTGCG-3′
  - R: 5′-TGCAAATGCTTGGCCCCCAGAG-3′;
- for *p21*
  - F: 5′-AGCAGCCGAGAGGTGTGAGC-3'
  - R: 5′-TGGACATGGTGCCTGTGGCTCCT-3'
- for *Mef2C*
  - F: 5′-TCCAAGTGCAGGTAACACAG-3'
  - R: 5′-TTGCCGTATCCATTCCCTG-3'
- for *Tbp*, used as housekeeping gene
  - F:5′-GGCGGTTTGGCTAGGTTT-3'
  - R: 5′-GGGTTATCTTCACACACCATG-3’.

### Chromatin immunoprecipitation (ChIP) assays

Nuclear lysates were prepared as previously described^19^. After determining the DNA concentrations, each chromatin sample was divided into aliquots corresponding to 150, 75 and 50 μg of DNA.

ChIP assays for PARP1, PAR and MyoD were carried out as previously described using 150 µg of chromatin for MyoD, and 75 µg for PARP1 and PAR. ChIP assays for H3K4me3 were performed using 50 µg of chromatin and Magna ChIP Protein A Magnetic Beads (Millipore) according to the manufacturer's instructions.

The following antibodies were used for immunoprecipitation: anti-PARP1 (ALX-210-302, Alexis Biochemicals); anti-PAR (4335-MC-100, Trevigen), anti-MyoD (G-1, sc-377460, Santa Cruz Biotechnology), anti H3K4me3 (07-473, Millipore), normal mouse IgG Antibody (12–371, Millipore) and normal rabbit IgG Antibody (12-370, Millipore).

qPCR analysis was performed in triplicate for each The following primer sets were used for qPCR analysis:

- for KvDMR1
  - F: 5′-GCACAAGTCGCAAGTCCGCG-3’
  - R: 5′-ATGGAGCCCAGCCGCGAAAG-3;
- for *myogenin* promoter
  - F: 5′-TGGCAGGAACAAGCCTTTTGCGA-3’
  - R: 5′-AGTCCGCTCATAGCCCGGGG-3′;
- for Rho promoter
  - F: 5′-TTGTCAACTGGAGCCATGTG-3'
  - R: 5′-GTGTCTCATCTTGTCTGCCC-3'
- for Igf2 DMR1
  - F: 5′-TGGAATGAGGAACATCACCA-3’
  - R: 5′-TCTATCCCTGGCTTTTCTGG-3’

### Statistical analysis

For chart making and for statistical analysis, comparisons were performed using parametric paired Student's t-test analysis with a confidence interval of 95%, using GraphPad Prism® graphic and statistical software. Statistical significance is shown as p < 0.05 (*) or p < 0.01 (**) or p < 0.001 (***).

## Supplementary information

Supplementary file1

## Data Availability

No datasets were generated or analysed during the current study.
