# Supplementary material for: Poly(ADP-ribose) Polymerase 1 (PARP1) restrains MyoD-dependent gene expression during muscle differentiation
Source: Sci Rep. 2020 Sep 15;10:15086. doi: 10.1038/s41598-020-72155-8 (PMC7493885; doi:10.1038/s41598-020-72155-8)
Supplement: Supplementary file 1 — Supplementary file1 [file 41598_2020_72155_MOESM1_ESM.docx]

**Supplementary Information**

**Poly(ADP-ribose) Polymerase 1 (PARP1) restrains MyoD-dependent gene expression during muscle differentiation**

**Francesca Matteini, Oriella Andresini, Stefano Petrai, Cecilia Battistelli, Marianna Nicoletta Rossi and Rossella Maione**

Supplementary Figures S1 – S14

Supplementary Methods


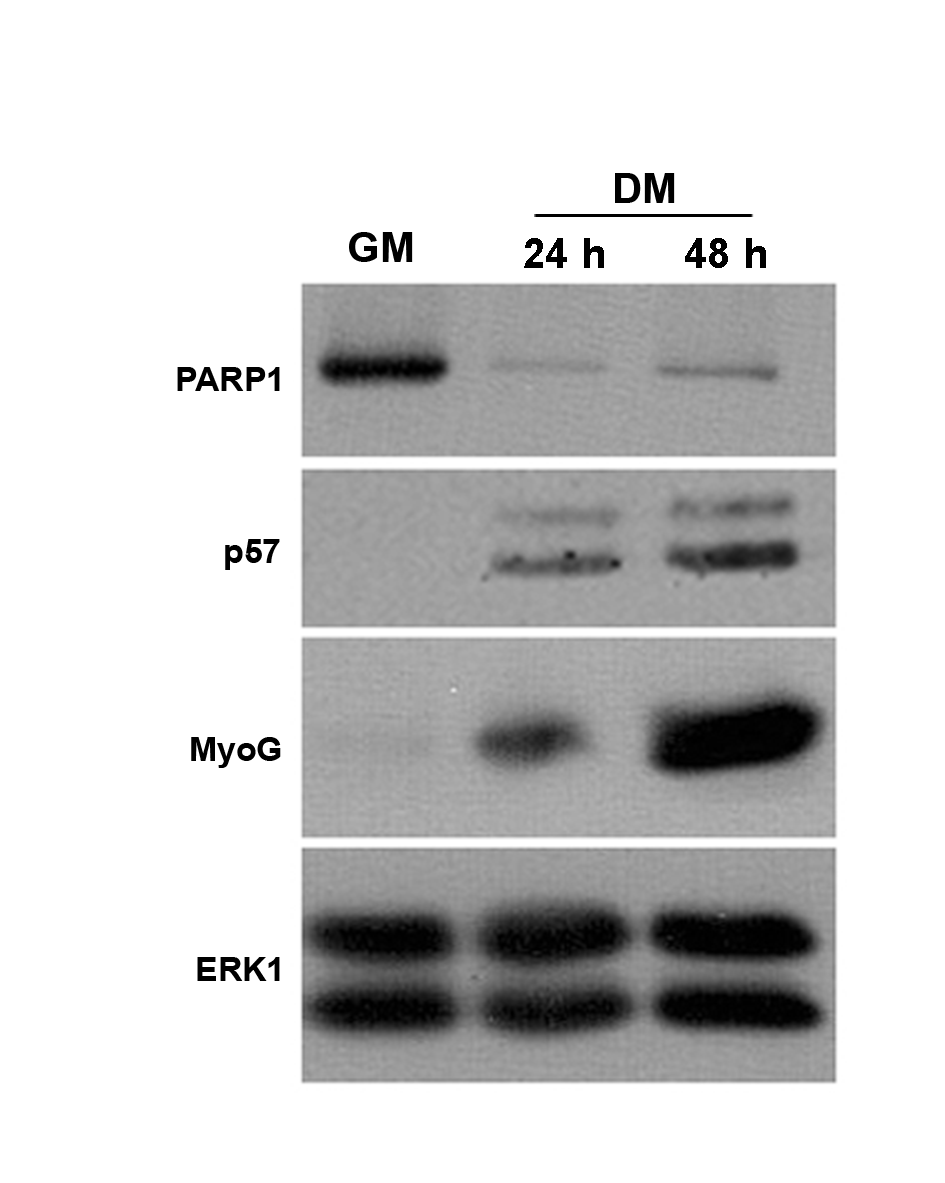


**Fig. S1:** *PARP1 protein levels drop during muscle differentiation*

Western blot analysis of PARP1 levels in undifferentiated C2.7 myoblasts proliferating in growth medium (GM) and in differentiated C2.7 myotubes cultured for 24 or 48 hours in differentiation medium (DM). p57 and myogenin (MyoG) were used to follow the differentiation process. ERK1 was used as an invariant control. Full length blots are presented in Fig. S12.


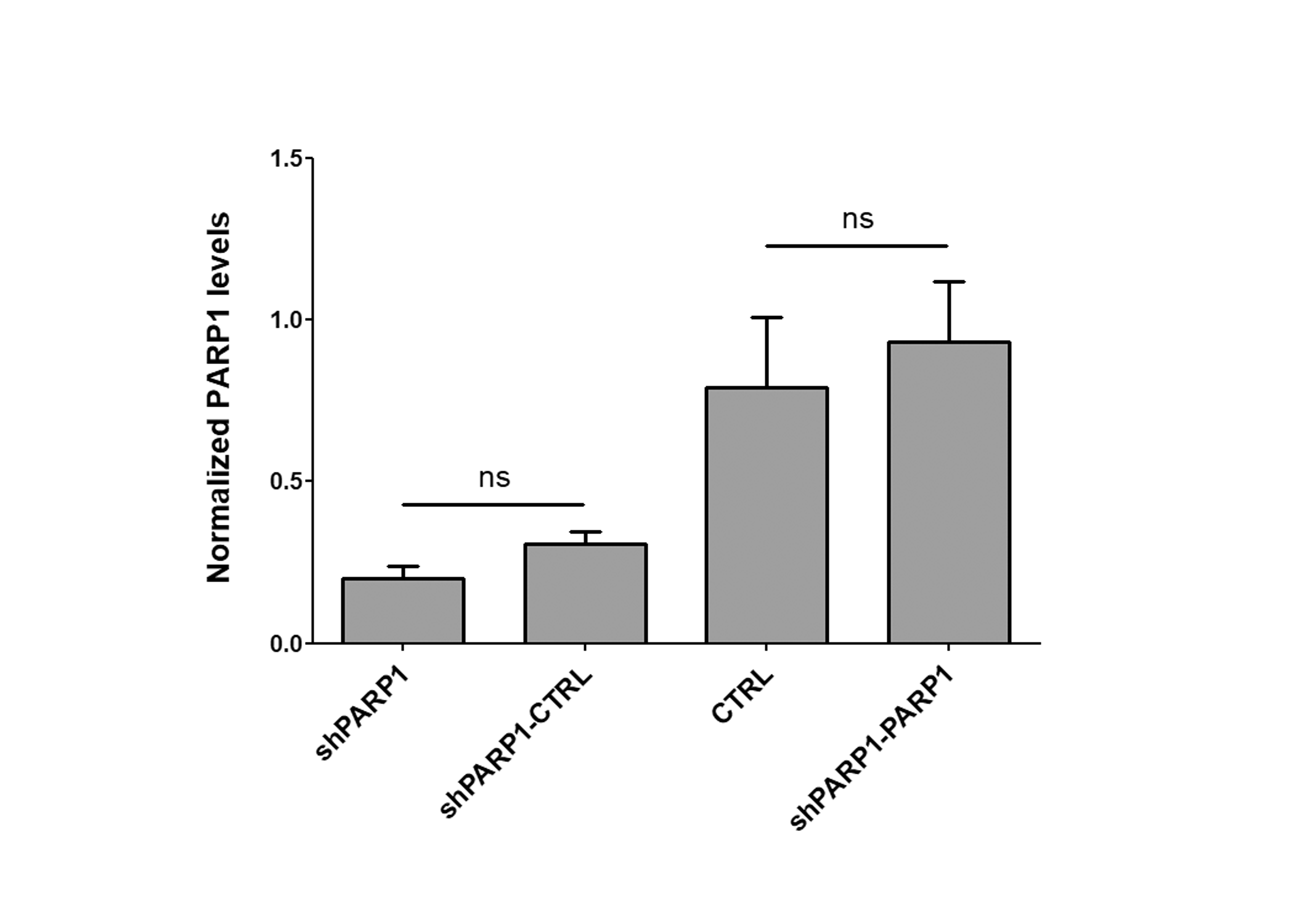


**Fig. S2:** *Comparison of PARP1 levels in the cell lines used in this work*

Quantification by densitometry of western blot analysis of PARP1 levels in C2.7 cells infected with the empty retrovirus (CTRL), C2.7 cells infected with the shPARP1 retrovirus (shPARP1), shPARP1 cells transfected with the puc19 empty vector (shPARP1-CTRL) and shPARP1 cells transfected with the puc19-PARP1 plasmid (shPARP1-PARP1). Columns were arranged in order to directly compare endogenous with exogenous PARP1 levels (CTRL and shPARP1-PARP1 respectively). PARP1 protein levels, normalized to the respective ERK1 levels, represent the mean ± SEM of two independent western blots. Statistical significance: ns stands for not significant.


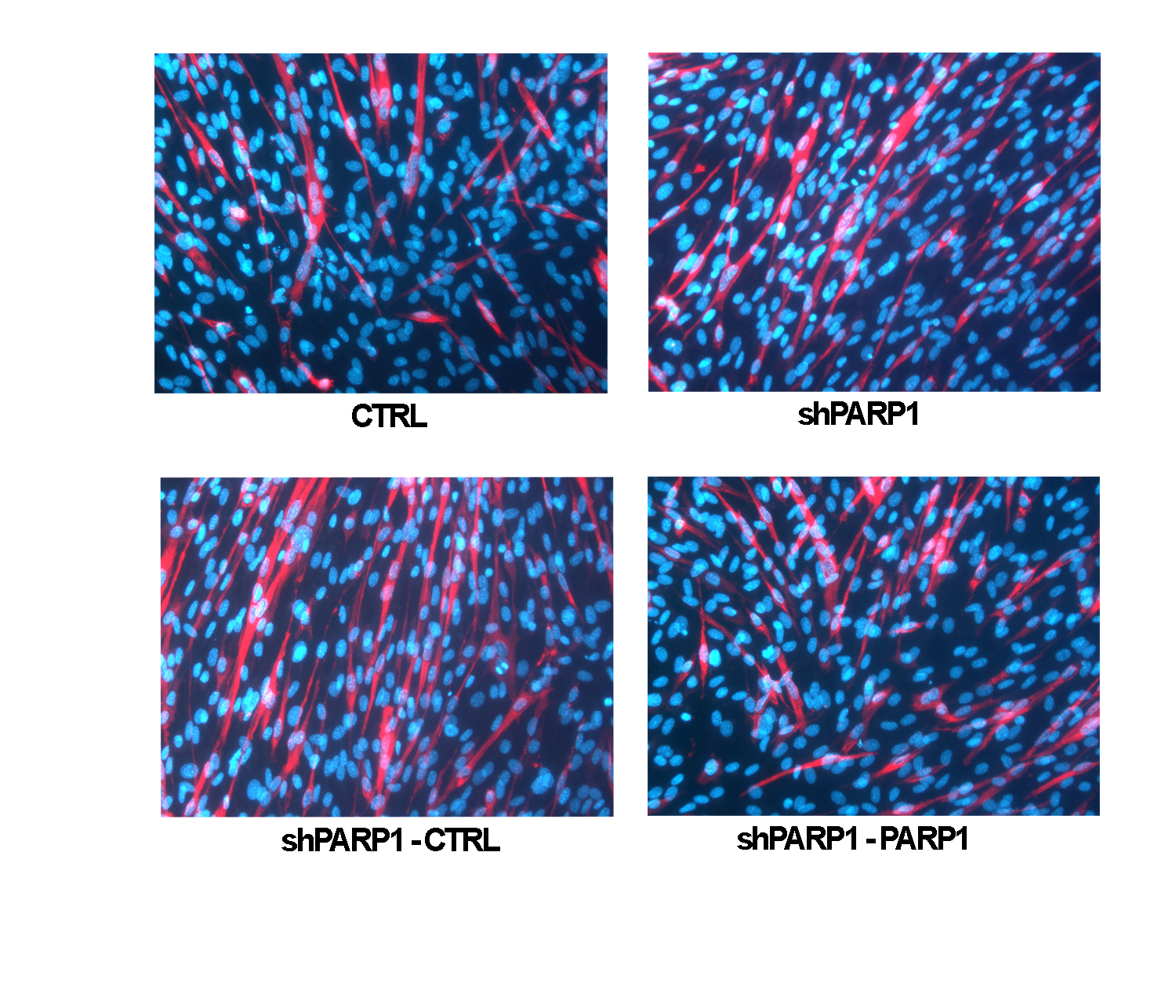


**Fig. S3:** *Representative images of the immunofluorescence analysis graphed in Figure 2C*

Immunofluorescence staining of MHC expression in C2.7 cells transduced with the empty retrovirus (CTRL), or with the shPARP1 retrovirus (shPARP1) and in shPARP1 cells stably transfected with the puc19 empty vector (shPARP1-CTRL) or with the puc19-PARP1 plasmid (shPARP1-PARP1). In red is shown MHC and in blue the DAPI-stained nuclei.


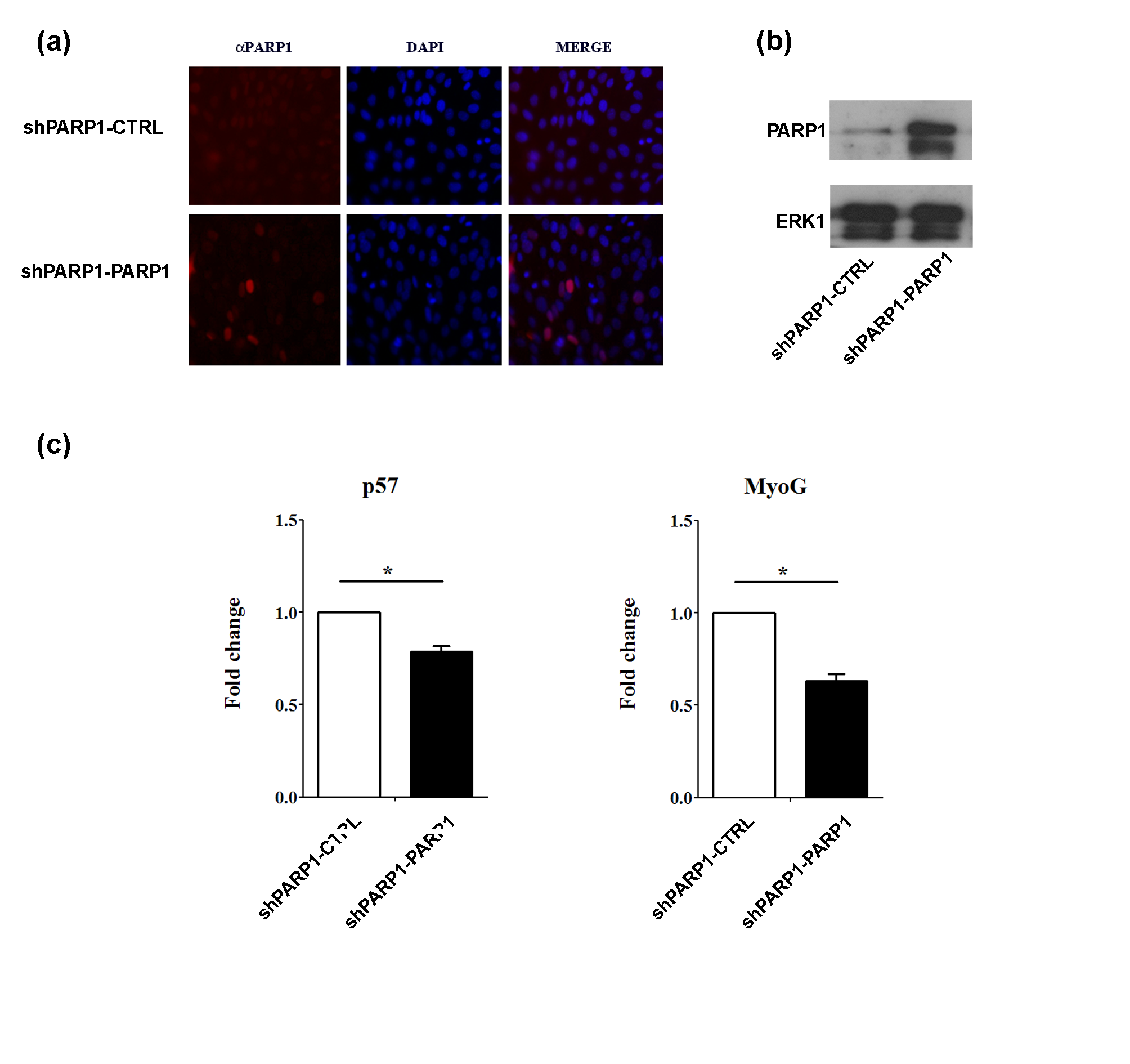


**Fig. S4:** *Transient over-expression of PARP1 inhibits p57 and myogenin induction*

**(a)** Immunofluorescence analysis of PARP1 expression in shPARP1 cells transiently transfected with the PARP1 expression vector (PARP1), compared to shPARP1 control cells (CTRL). In red is shown PARP1 and in blue the DAPI-stained nuclei. The merged images combining the fluorescence for PARP1 and DAPI are also shown. Images were acquired by a Nikon Microphot – FXA microscope, using a 20X objective. **(b)** Western blot analysis of PARP1 levels in the above mentioned cells. ERK1 was used as an invariant control. **(c)** RT-qPCR analysis of *p57* and *myogenin* (MyoG) in the above-mentioned cells, cultured for 24 hours in differentiation medium. Expression levels, relative to those of Tbp RNA and expressed as fold change respect to the control, are reported as the mean ± SEM of two independent experiments. Statistical significance: p-value<0,05 (*).


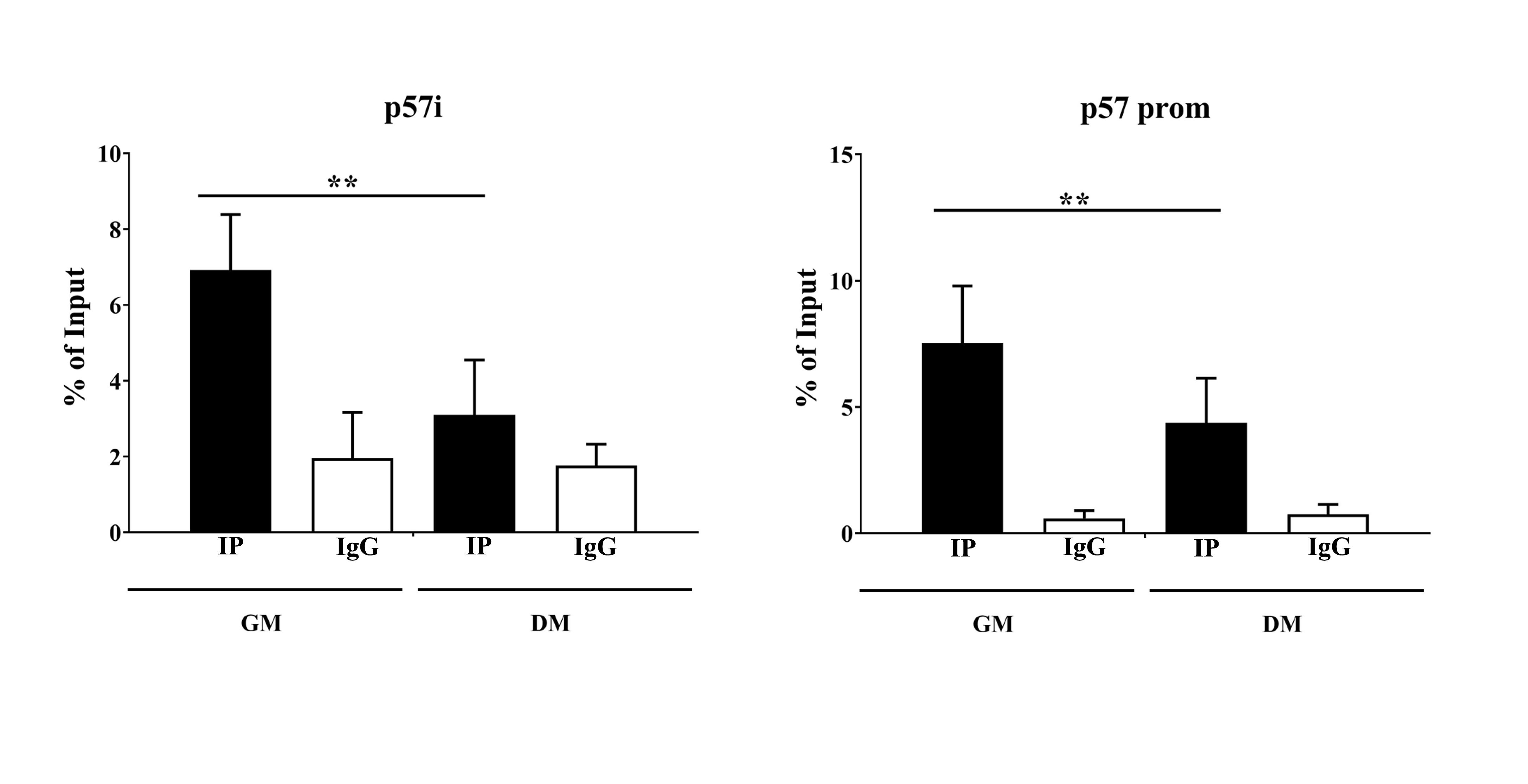


**Fig. S5:** *PARP1 interacts with the intragenic p57 regulatory region and with p57 promoter*

ChIP-qPCR analysis for PARP1 binding to a previously described *p57* intragenic region (p57i) and to *p57* promoter (p57 prom), during C2.7 cell differentiation. Samples were prepared from undifferentiated cells proliferating in growth medium (GM), and from differentiated cells kept for 24 hours in differentiation medium (DM) and immunoprecipitated either with PARP1 antibody (IP samples) or with control IgG. Protein binding is expressed as percentage of input and represents the mean ± SEM of four independent experiments. Statistical significance: p-value <0,01 (**)**.**


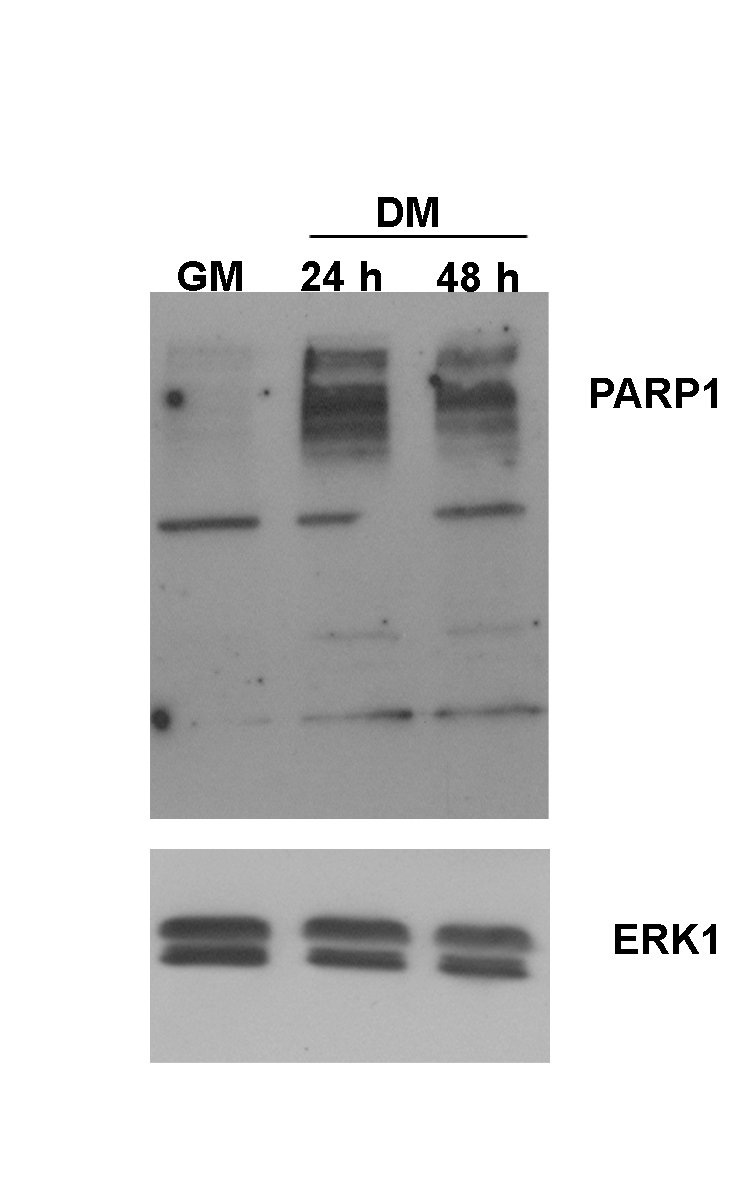


**Fig. S6:** *PAR levels increase during muscle differentiation*

Western blot analysis of PARylated proteins in undifferentiated C2.7 myoblasts proliferating in growth medium (GM) and in differentiated C2.7 myotubes cultured for 24 or 48 hours in differentiation medium (DM 24 h and DM 48 h, respectively) and lysed in Laemli buffer. PARylated proteins were detected with the anti-PAR antibody. ERK1 was used as an invariant control. Full length blots are presented in Fig. S14.


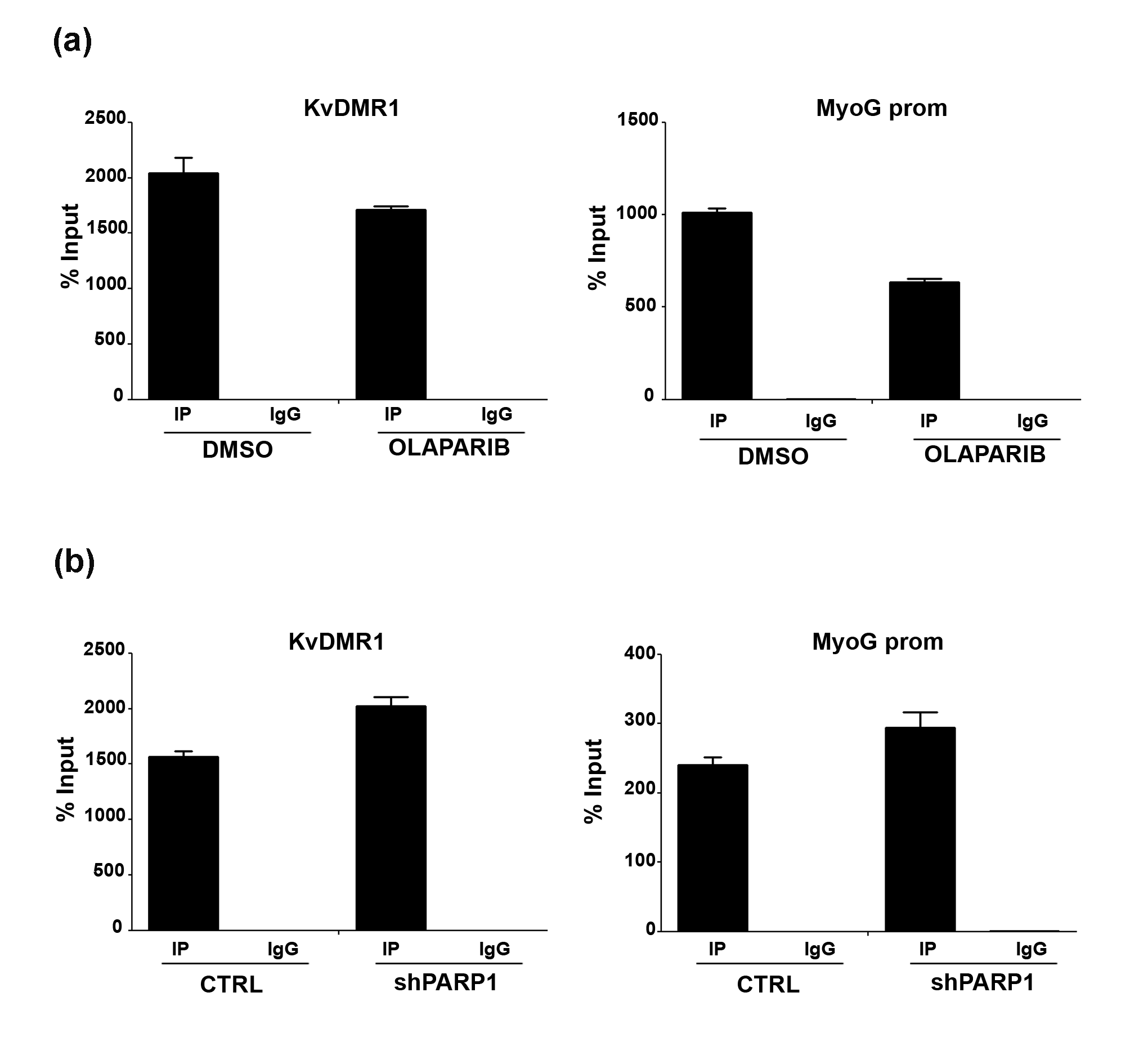


**Fig. S7** *Olaparib-treatment decreases, whereas PARP1-depletion increases the levels of H3K4me3 at p57 and myogenin regulatory regions*

ChIP-qPCR analysis for H3K4me3 association to KvDMR1 and *myogenin* promoter: (**A**) in C2 cells differentiated for 24 hours in the presence of Olaparib or the control vehicle DMSO; (**B**) in C2.7 cells stably transduced with shPARP1 or with the empty vector (CTRL), 24 hours after the shift to differentiation medium. Protein binding is expressed as percentage of input and represents the mean ± SEM of three technical replicates of a representative experiment for each of the two experimental settings.


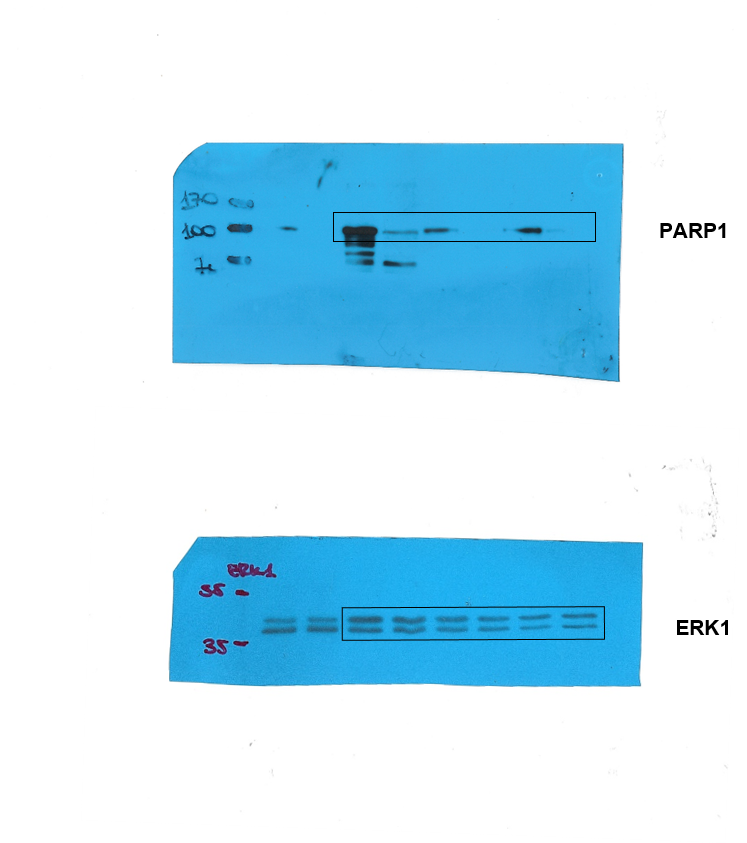


**Fig. S8:** *Full length blots of Figure 1*

Boxes indicate cropped regions


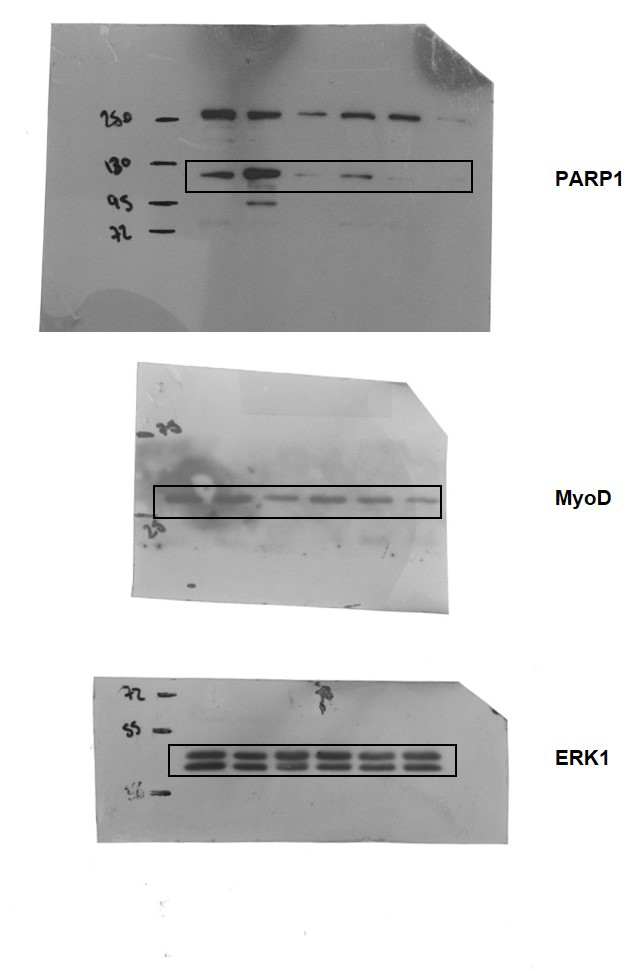


**Fig. S9:** *Full length blots of Figure 2A*

Boxes indicate cropped regions


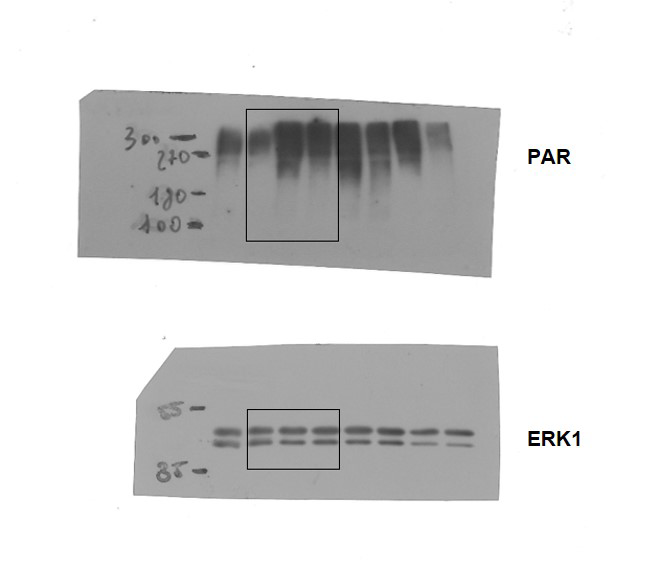


**Fig. S10:** *Full length blots of Figure5a*

Boxes indicate cropped regions


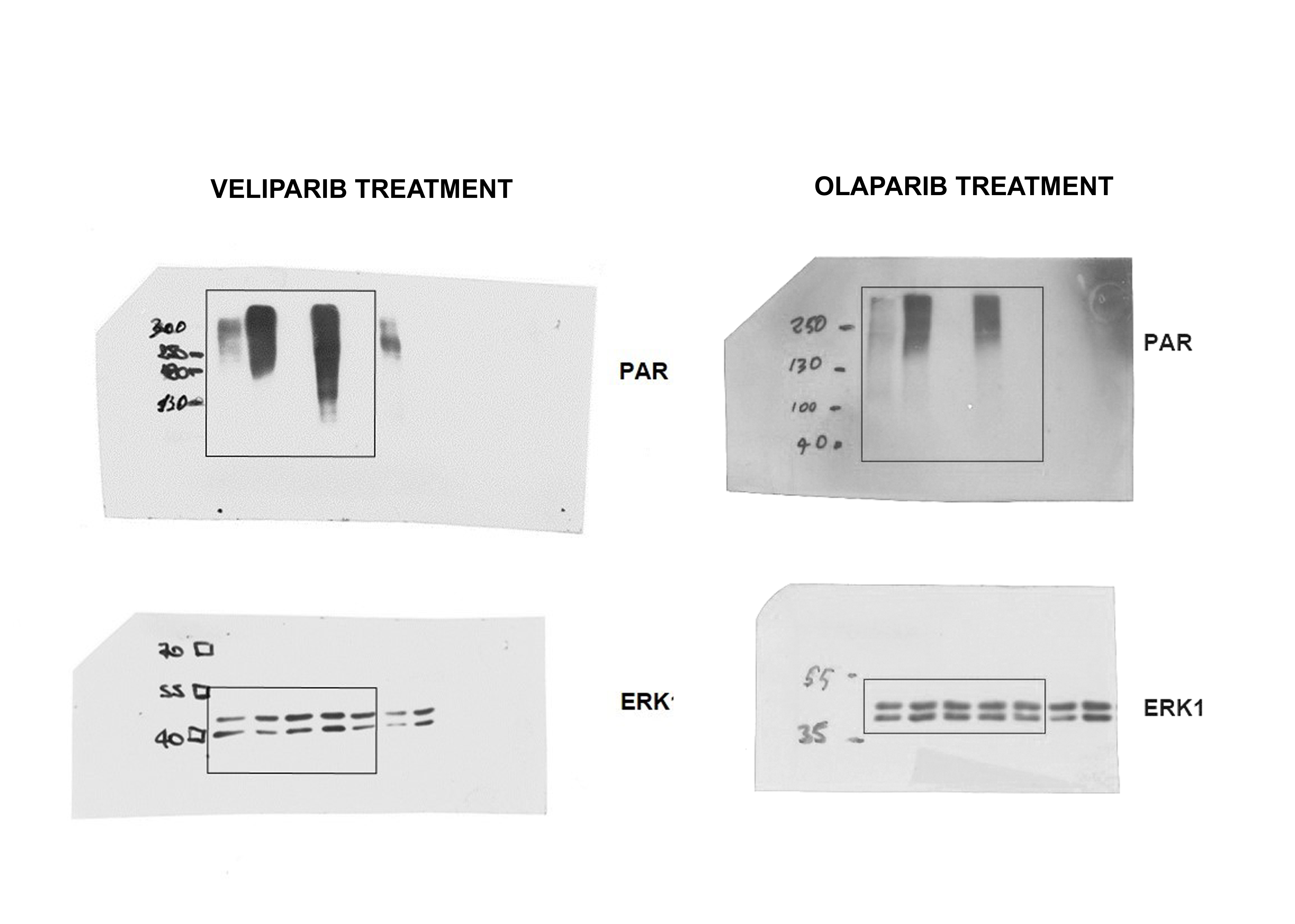


**Fig. S11:** *Full length blots of Figure 6*

Boxes indicate cropped regions


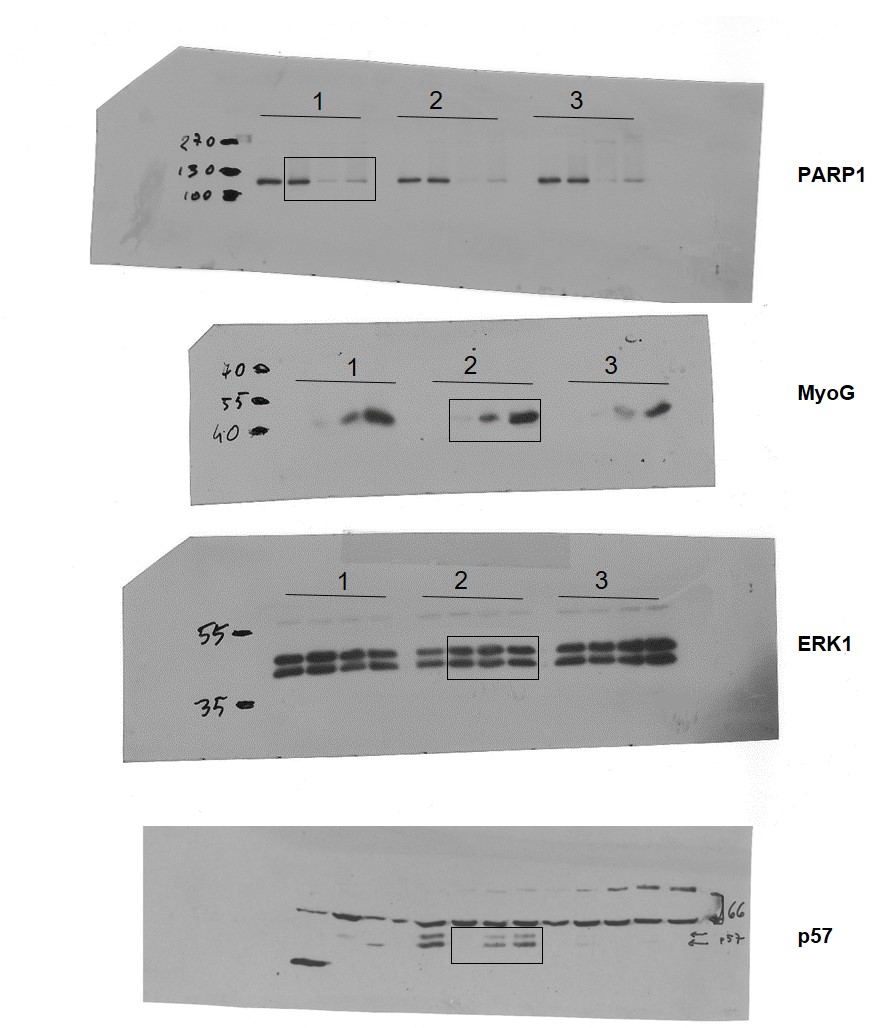


**Fig. S12:** *Full length blots of Figure S1*

Boxes indicate cropped regions. Groups 1, 2 and 3 indicate three biological replicates of the time course experiment, loaded on the same gel and analyzed for PARP1, Myogenin and ERK1. p57 western blot was performed on a different gel, due to the damaging of the filter. However, in light of the well established and reproducible kinetics of p57 up-regulation, reported in our previous publications and used here just to follow the differentiation process, similarly to Myogenin, we believe that in this case the use of a different gel is justifiable.


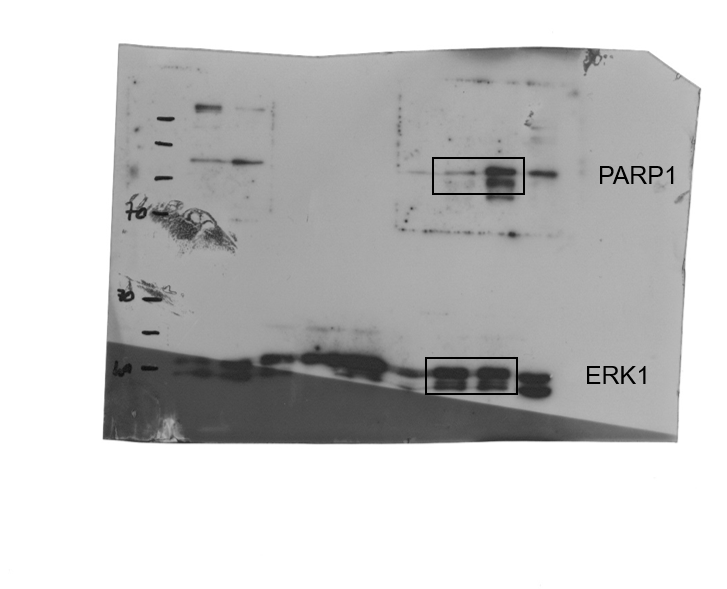


**Fig. S13:** *Full length blots of Figure S4b*

Boxes indicate cropped regions


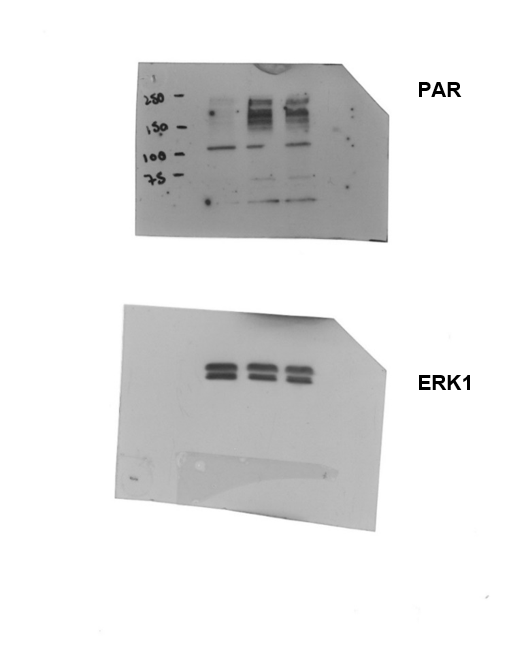


**Fig. S14:** *Full length blots of Figure S6*

***Supplementary Methods***

*Transient transfection*

Transient re-expression of PARP1 was obtained by transfecting either the puc19-PARP1 plasmid or the empty vector puc19 into shPARP1 C2.7 cells. 24 hours after transfection cells were shifted to differentiation medium and collected 48 hours later for RNA analysis and for testing PARP1 expression by immunofluorescence staining.

*Immunofluorescence staining*

Cells were fixed with 1% formaldehyde for 20 minutes at room temperature and the reaction was stopped by 0,125 M cold glycine for 15 minutes. Fixed cells were incubated with the F1-23 anti-PARP1 antibody (Alexis) diluted 1:50 in 3% BSA/PBS or with the undiluted MF20 hybridoma supernatant, specific for Myosin Heavy Chain (MHC) and then with the rhodamine-conjugated anti-mouse IgG (Cappel) diluted 1:150 in 3% BSA/PBS. To counterstain nuclei, cells were incubated with 4′,6-Diamidine-2′-phenylindole dihydrochloride (DAPI) for 5 min at room temperature. Immunostained cells were analysed on Nikon microphot FXA equipped with a 10x objective.

*Primers sets for ChIP assays*

For p57i:

F: 5′-AAC TTC CAG CAG GAT GTG CC-3′
R: 5′-CAT CCA CTG CAG ACG ACC AG-3′

For p57 prom:

F: 5′ACT GAG AGC-AAG CGA ACAGG-3′
R: 5′ACC TGG CTG ATT GGT GAT GG-3′
